# Cyclophilin A regulates secretion of tumour-derived extracellular vesicles

**DOI:** 10.1016/j.tranon.2021.101112

**Published:** 2021-05-10

**Authors:** Yunjie Wu, Kieran Brennan, Alfonso Blanco Fernández, Margaret M. Mc Gee

**Affiliations:** a UCD School of Biomolecular & Biomedical Science, Conway Institute, University College Dublin (UCD), Belfield, Dublin 4, Ireland; b Flow Cytometry Core Technology, Conway Institute, University College Dublin (UCD), Belfield, Dublin 4, Ireland

**Keywords:** Extracellular vesicles, Cyclophilin A, Haematopoietic malignancies, Pro-inflammatory

## Abstract

Extracellular Vesicles (EVs) are a heterogenous population of particles that play an important role in cell-cell communication in physiological and pathophysiological situations. In this study we reveal that the peptidyl prolyl isomerase Cyclophilin A (CypA) is enriched in cancer-derived EVs from a range of haematopoietic malignancies. CypA-enriched blood cancer EVs were taken up by normal monocytes independent of EV surface trypsin-sensitive proteins and potently stimulated pro-inflammatory MMP9 and IL-6 secretion. Further characterisation revealed that CypA is intravesicular, however, it is not present in all EVs derived from the haematopoietic cells, instead, it is predominantly located in high density EVs with a range of 1.15–1.18 g/ml. Furthermore, loss of CypA expression in haematological cancer cells attenuates high density EV-induced pro-inflammatory MMP9 and IL-6 secretion from monocytes. Mechanistically, we reveal that homozygous loss or siRNA knockdown of CypA expression significantly reduced the secretion of EVs in the range of 100–200 nm from blood cancer cells under normal and hypoxic conditions. Overall, this work reveals a novel role for CypA in cancer cell EV biogenesis.

## Highlights

• Blood cancer EVs stimulate pro-inflammatory immune signals.
• Cyclophilin A is a core EV protein and is localised in high density blood cancer derived EVs.
• Cyclophilin A regulates biogenesis and/or release of EVs with a diameter of 100 to 200 nm.

## Introduction

Extracellular Vesicles (EVs) are small spheres, ranging in size from 50 to 5000 nm, enclosed by a lipid bilayer that are released from cells [1], and mediate local and systemic cell-cell communication via the horizontal transfer of functional protein, DNA and RNA to recipient cells [2]. EVs are present in all body fluids including urine, breast milk and blood [3]. EVs are characterized as either exosomes, microvesicles, and apoptotic bodies based on their size and biogenesis pathway. Microvesicles and apoptotic bodies are produced by outward budding directly from the cell membrane whereas exosomes are produced via the endosomal pathway, and are released into the extracellular space when the multivesicular bodies (MVBs) fuse with the plasma membrane [4]. EVs secreted from cells interact with recipient cells to induce changes in their physiology. While EV-induced signalling can occur at the cell surface via protein-protein interactions, they have been widely reported to deliver cargo to the recipient cells following internalisation which occurs through a variety of receptor-dependant and -independent processes [5].

The secretion of EVs and EV-mediated communication is an evolutionarily conserved phenomenon [6] and has been associated with numerous diseases. Circulating levels of EVs of different cellular origin are increased in cardiovascular disease [7] and EVs participate in the spreading of misfolded proteins in neurodegenerative disorders [8], while they can suppress the immune response and facilitate angiogenesis and pre-metastatic niche formation thereby promoting tumori-genesis [9].

Tumour-derived EVs, including blood cancer-derived EVs, mediate intercellular communication between tumour cells and normal cells within the tumour microenvironment and can facilitate pre-metastatic niche formation [10]. Thus, it is hypothesized that cancer metastasis could be restricted by interfering with EV-mediated intercellular communication within the pre-metastatic niche. EV signalling within the tumour microenvironment remains poorly understood, therefore, a better comprehension of EV heterogeneity and function is essential, and will reveal strategies to target EVs in cancer therapy [11].

Peptidyl prolyl isomerases (PPIases) are an enzymatic family comprised of cyclophilins, FK-506 binding proteins and parvulins [12] that catalyse the *cis-trans* isomerisation of peptidyl proline bonds and are involved in protein folding [13], trafficking and signal transduction [14]. Cyclophilins were originally identified as receptors for the immunosuppressive drug, cyclosporin A [15]. Amongst cyclophilins, CypA has been shown to regulate many biological processes including protein complex stabilization and cell division [16]. Furthermore, CypA can function as a molecular chaperone independent of its PPIase activity [17].

Previous studies demonstrate that CypA is upregulated in tumour cells and is a key determinant of cancer transformation, metastasis, and chemoresistance [18,19]. CypA is also important for cancer cell division [20]. Moreover, CypA plays an important role during cytokinesis [20] and viral budding from plasma membrane [21], processes that are topologically similar to EV biogenesis such as membrane constriction and abscission that occurs during intraluminal vesicle (ILV) formation and microvesicle budding. However, to date a role for CypA on EV biogenesis has not been investigated.

In this study, we demonstrate that blood cancer EVs are taken up by monocytes and stimulate pro-metastatic inflammatory signals. Furthermore, we investigated the influence of CypA on blood cancer EV secretion and communication with immune cells. CypA is found to be enriched in high density EVs released from a range of blood cancer cell lines. CypA-enriched high density EVs promote pro-inflammatory signals in recipient immune cells and loss of CypA expression attenuates EV secretion and pro-inflammatory signals. Overall, this study reveals a novel function for CypA in secretion of tumour-derived EVs within the tumour microenvironment.

## Materials and methods

### Antibodies and reagents

The primary antibodies: anti-Cyclophilin A (1:1000 dilution, Abcam, ab126738), anti-CD147 (1:1000, ThermoFisher, MA1–19,201), anti-HSP70 (1:3000, Santa Cruz, sc-66,048), anti-TSG101 (1:1000 dilution, Abcam, ab125011), anti-HSP90B1 (1:1000 dilution, Cusabio, CSB-PA10887A0Rb), anti-GAPDH (1:500 dilution, EMD Millipore, MAB374), anti-Rab7 (1:1000 dilution, Cell signaling Technology, 9367), anti-Rab11 (1:250 dilution, ThermoFisher, 71–5300).

The secondary antibodies: anti-Rabbit IgG-DyLight 800 (1:10,000 dilution, ThermoFisher, SA5–35,571), anti-Mouse IgG-DyLight 680 (1:10,000 dilution, ThermoFisher, 35,519)

Reagents: Trypsin (Pierce Trypsin Protease, MS Grade, ThermoFisher, 90,057), Propidium Iodide (ThermoFisher, P1304MP), 5-(and −6)-Carboxyfluorescein Diacetate, Succinimidyl Ester, mixed isomers (5(6)-CFDA, SE) (ThermoFisher, C1157)

### Cell culture

The human B lymphocytic leukaemia cell line HG3 and I83 (a kind gift from Dr. Tony McElligott, St. James's Hospital and Trinity College Dublin, Ireland), human myelogenous leukaemia cell line K562 (ATCC:CCL-243), myeloma cell line U266 (ATCC: TIB-196), T lymphocyte cell line Jurkat WT and CypA-/- [22], and human monocytic cell line THP-1 (ATCC: TIB-202) were grown in RPMI 1640 medium (Gibco) with 10% foetal bovine serum (FBS) (Gibco) and 1% Penicillin-Streptomycin (Gibco). RPMI 1640 medium with 1% Penicillin-Streptomycin only was used as serum-free medium. Cells were cultured at 37 °C in 5% CO_2_ humidified incubator and passaged every 2–3 days at 70%−80% confluence.

### EV isolation and iodixanol density gradient separation

**Fig. 1:**
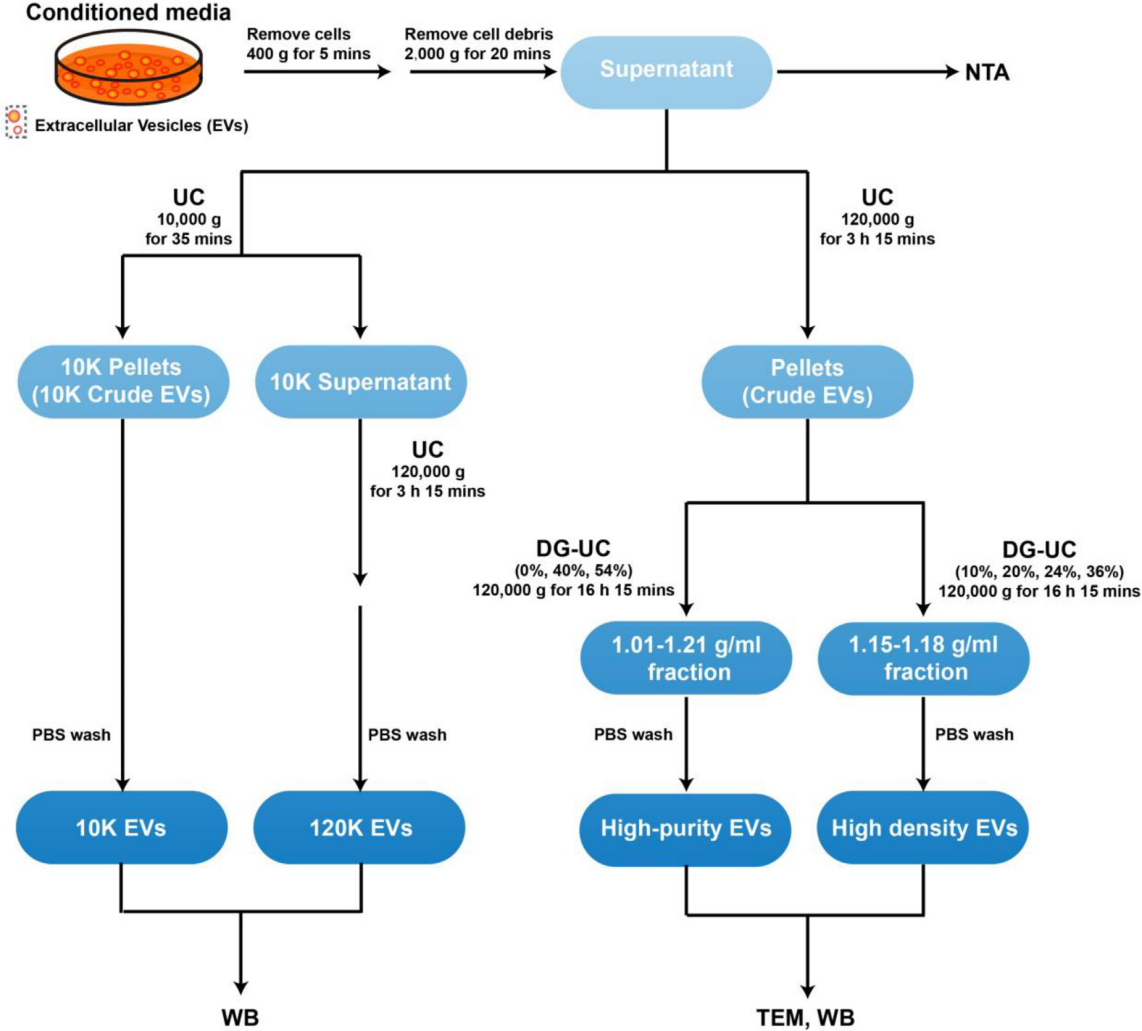
Streamlined workflow of different methods for EV isolation. Conditioned media was collected after 48 h serum-free media treatment with different blood cancer cell lines (1 × 10^6^/ml). Cells and cell debris were removed from conditioned media by centrifuging at 400 g/ 5 min and 2000 g/ 20 min respectively. 10 K and 120 K crude pellets were enriched from conditioned media by differential ultracentrifugation at 10,000 g and 120,000 g respectively. Each pellet was resuspended in PBS and ultracentrifuged again at 120,000 g to remove soluble contaminants. 10 K and 120 K EV pellets were analysed by western blotting. Alternatively, crude EVs were isolated from conditioned media by ultracentrifugation (UC) and were further processed by ultracentrifugation with different settings of density gradient (DG-UC) at 120,000 g for isolation of high-purity and high density (HD) EVs.High-purity and HD EVs were analyzed by western blotting (WB) and Transmission electron microscopy (TEM).

HG3, I83, Jurkat, K562, U266 cells were grown in T175 cell culture flasks and cell viability was more than 90% before 48 h serum-free medium treatment. Conditioned media from each cell line (1 × 10^6^ cells/ml) grown in 145/20 mm cell culture dishes after 48 h serum-free medium treatment was used for EV isolation. Cell number and cell viability at the time of EV harvesting were checked by haemocytometer and PI-staining flow cytometry respectively. Cells were removed by centrifugation at 400 g for 5 min, and then cell debris were removed by centrifugation at 2000 g for 20 min. An overview of EV isolation methods is provided in Fig 1.

#### Ultracentrifugation (UC)

For 10 K and 120 K EV isolation, the supernatant was processed with an extra step after 2000 g centrifugation by centrifuging at 10,000 g_avg_ for 35 min to get 10 K pellets and then 120 K pellets were collected at 120,000 g_avg_ (SW32Ti, k-factor 204, Beckman Coulter) for 3 h 15 min (centrifugation durations adjusted based on a “50 nm cut-off size” as described in the paper [23]). The 10 K and 120 K pellets were subsequently washed in PBS with ultracentrifugation at 120,000 g_avg_ for 3 h 15 min. The 10 K and 120 K EVs were resuspended in PBS and were freshly processed.

#### Floatation density gradient (DG-UC)

For high-purity EV isolation, the EV-enriched pellet was collected at 120,000 g_avg_ (SW32Ti, k-factor 204, Beckman Coulter) for 3 h 15 min and resuspended in particle-free PBS. The EV-enriched pellet was further isolated by flotation on iodixanol density gradients. A total of 300 µl EV-PBS sample was mixed with 2.7 ml 60% iodixanol and laid at the bottom of the tube, and 3 ml layer of 40% iodixanol, and PBS were subsequently overlaid forming a discontinuous gradient. Samples were performed with ultracentrifugation at 120,000 g_avg_ (SW41Ti, k-factor 143.9, Beckman Coulter, stop without brake) for 16 h 15 min. The EV layer present between PBS and 40% iodixanol was transferred to a new tube and washed in PBS with ultracentrifugation at 120,000 g_avg_ for 3 h 15 min. The highly purified EVs were resuspended in PBS and were processed fresh.

#### Gradient fractionation (DG-UC)

For EV fractionation, the EV-enriched pellet was further fractionated by flotation on iodixanol density gradients. A total of 1.2 ml of EV-PBS sample was mixed with 1.8 ml 60% iodixanol and laid at the bottom of the tube, and 3 ml layer of 24%, 20%, 10% iodixanol were subsequently overlaid forming a discontinuous gradient. Samples were performed with ultracentrifugation at 120,000 g_avg_ (SW41Ti, k-factor 143.9, Beckman Coulter, stop without brake) for 16 h 15 min. Each 100 µl fraction was collected from the top to bottom of the tube and distributed into 96-well plate for absorbence measurement at 340 nm by multi-well microplate spectrophotometer and density was calculated from an Iodixanol standard curve. Next, the fractions with densities <1.06 g/ml, 1.06 - 1.09 g/ml, 1.09 - 1.12 g/ml, 1.12–1.15 g/ml, 1.15–1.18 g/ml and 1.18–1.21 g/ml were pooled to form six individual fractions respectively. The six fractions were then diluted and washed in PBS with ultracentrifugation at 120,000 g_avg_ for 3 h 15 min (SW41Ti, k-factor 143.9, Beckman Coulter). All centrifugation was performed at 10 °C. The fractionated EV pellets were resuspended in PBS and were freshly processed.

### Flow cytometric analysis

#### Cell viability quantification

The live cells were harvested and resuspended before being washed with PBS and centrifuged three times at 400 g for 5 min. The resuspended cell solutions were stained with 10 μg/mL propidium iodide (PI) (The ratio of cell number to PI quantity was 10 μg PI per 10^6^ cells). Next, the stained solutions were mixed gently and incubated for 1 min in the dark. PI was excited by using a 488 nm laser and its fluorescence was collected by using FL-2 channel (575/25 band pass filter) in BD AccuriTM C6. The stop count was set at 10,000 events on the cell singlets gate generated in a bivariate histogram of forward scatter area versus high. The percentage of cell viability was determined by measuring the negative population (established with the unstained control). The data were obtained using BD AccuriTM C6 software v1.0 (BD, USA) and were analysed using FlowJoTM v.10 (BD, USA). The volume of the sample is measured automatically by the instrument which was properly calibrated under manufacture specifications.

#### EV uptake confirmation

After EV isolation by ultracentrifugation, 20 mM 5(6)-carboxyfluorescein diacetate (CFDA) was used to label EVs for 1 h at 37 °C. EVs were further centrifuged by density gradient to remove excess dye, soluble protein, and protein aggregates. Labelled EVs were collected and incubated with THP-1 cells for 24 h, the cells were collected and washed at least three times with PBS. CFDA fluorescence from cells was measured using FL-1 channel (533/30 band pass filter) in BD Accuri™ C6. The data were obtained using BD Accuri™ C6 software v1.0 (BD, USA) and were analysed using FlowJo™ v10 (BD, USA).

#### CD147 staining and EV detection

Flow cytometry analysis was performed on the Beckman Coulter CytoFLEX LX Flow Cytometer. 0.04 nm filtered water was used as Sheath fluid. For daily calibration of the flow cytometer CytoFLEX Daily QC and CytoFLEX Daily IR QC Fluorospheres beads as per manufacturer specifications, followed by Apogee Mix Silica (Si) and polystyrene (PS) beads (Apogee Flow Systems Ltd. Hertfordshire, UK) were used in sizes of PS 80 nm, PS 110 nm, Si 180 nm, Si 240 nm, Si 300 nm, PS 500 nm, Si 590 nm, Si 880 nm and Si 1300 nm. Extensive cleaning cycles have been constantly done. Compensation was not needed. Fluorescent gain values were established by a Gaintration process, meanwhile the gain of the scatters (from the blue, red and violet lasers) and threshold was established with the help of the Apogee Mix beads (VSSC gain=300; VSSC—H threshold=5500). Events were gated on the VSSC-width log x VSSC—H log cytogram to remove EV aggregates (singlet gate), A rectangular gate was set on the VSSC—H log x RSSC—H log cytogram containing the 80 nm and 500 nm bead populations and defined as ‘PS beads 80 nm–500 nm gate’ followed by a “stable time gate” set on the time histogram in order to identify the microparticle region (Fig. S9). To avoid swarm effects each was serially diluted from 1:2 to 1:500 to achieve an event count of 5000 events/s and measured with a flow rate of 10 μl/min prior to antibody labelling. EVs were labelled with 0.1ml anti-CD147-APC (MEM-M6/1, Thermo Scientific) in 100 µl PBS for 30 min on ice in the dark. Surface CD147 expression was measured using the 638 nm laser and the 660/20 band pass filter. To avoid false positive events, all antibodies were centrifuged before use at 20,000 g for 20 min and were run in PBS alone to ensure antibody aggregates were not present. To avoid carry‐over effects between each sample measurement, a washing step with filtered double distilled water for 1 min at an increased flow rate of 60 μl/min was performed. EV lysis was performed by incubating PBS-diluted EVs in 0.1% Triton™ X-100 for 30 min at room temperature. The data were obtained using CytExpert v2.3 (Beckman Coulter, USA) and were analysed using FCS Express™ v6 (De Novo Software, USA).

### Transmission electron microscopy

10 μl of resuspended EV samples in PBS was placed onto the shiny side of the TEM grids (formvar carbon coated 200 mesh copper grid) and incubated for 30 min. The excess fluid was removed by blotting with a filter paper. The grids were rinsed by dipping in PBS 3 times and dried by a filter paper. The samples were fixed by adding 2.5% glutaraldehyde and were incubated for 10 min at room temperature before washing the grids 5 times with distilled water. The 2% uranyl acetate was added onto the grids and incubated for 15 min at room temperature. The grids were rinsed quickly with ice-cold 1.8% methyl cellulose and 0.4% uranyl acetate (MC/UA). The samples were embedded by adding MC/UA for 10 min on ice. The grids were air dried at room temperature and examined by TEM (FEI Tecnai™ 120 kV transmission electron microscope).

### Nanoparticle tracking analysis

The size and particle concentration of all EV preparations was measured by a NanoSight NS300 system (Malvern Technologies, Malvern, UK) combined with a 488 nm laser and a high sensitivity scientific CMOS camera. Samples were diluted with an appropriate concentration in particle-free PBS (Gibco, Waltham, MA, USA) based on manufacturer's recommendations. Samples were analysed under constant flow conditions (flow rate = 50) at 25 °C and 15 captures for 60 s/capture were analysed using NTA3.2 software with a detection threshold of 5 and a bin size of 2.

### Cell transfection with siRNA and collection of conditioned media

K562 cells were transfected with pooled CypA siRNA or control scrambled siRNA by Amaxa Biosystems-Nucleofector® II. The optimum ratio of cells and CypA siRNA for nucleofection was determined to be 10^6^cells transfected with 4 μl of 5 nM siRNA stock. The cells were harvested after 24 h cell culture and counted by a haemocytometer. 1 × 10^6^ cells/ml of K562 cells were incubated with serum-free medium in a 6-well plate for 48 h. Conditioned media collected after 48 h was centrifuged at 400 g for 5 min following by 2000 g for 20 min to remove cells and cell debris. EVs in the supernatant were measured by nanoparticle tracking analysis.

### Hypoxic exposure in vitro

Cells seeded in 6-well plate were exposed to defined atmospheric conditions in environmental chamber (Coy Laboratories, Grass Lake, MI). Experimental atmospheres were designed to mimic the levels of O_2_ under hypoxic condition. Hypoxia in vitro was defined as 1% O_2_. At the end of experimental exposure, cell media and cell lysates were harvested within the chambers to prevent the confounding effects of reoxygenation. Cell media were used for nanoparticle tracking analysis, while cell lysates were used for confirmation of hypoxia.

### Protease treatment

Isolated EVs were incubated with 2–20 µg/ml trypsin in PBS for 30 min at 37 °C. The proteinase activity was then inhibited by adding 5 mM phenylmethylsulfonyl fluoride (PMSF) for 10 min at room temperature. All protease-treated EVs were immediately processed.

### Protein determination and Western blotting

Cell or EV proteins were extracted in RIPA lysis buffer supplemented with 1 mM PMSF, 1 μg/ml aprotinin, 1 μg/ml leupeptin, 1 μg/ml pepstatin A, 100 μM Sodium orthovanadate after washing samples twice with ice-cold PBS. The lysed samples were incubated on ice for 20 min and centrifuged at 14,000 RPM for 30 min, 4 °C. EV protein content was measured using a bicinchoninic acid (BCA) protein assay kit (Thermo Fisher Scientific) and cell protein content was measured using BCA or Bradford protein assay (Sigma) according to manufacturer's protocol.

For western blotting, protein samples were prepared in PBS with SDS loading buffer and followed by heating to 95 °C for 10 min. Proteins were separated on 5%−12% SDS-PAGE gels using a Bio-Rad Mini-Protean II gel system and transferred to 0.45 µm nitrocellulose membrane using a mini-Protean II blotting system at 110 V constant voltage for 70 min. Then membranes were blocked with TBS blocking buffer (5% bovine serum albumin in TBS) for 1 h at RT and incubated with primary antibodies shown above in TBS blocking buffer at 4 °C overnight. The membranes were washed three times with 0.1% TBS-Tween and then incubated with IRDye800-conjugated goat anti-rabbit IgG or IRDye680-conjugated goat anti-mouse IgG secondary antibodies (LI-COR Biosciences) diluted 1:10,000 in TBS blocking buffer for 2 h at RT. Next, the blots were washed six times for 5 min with TBS-Tween, visualized by Odyssey Infrared Imaging System (LI-COR Biosciences) with both 700- and 800-nm channels. The proteins were analysed with Image Studio v.5.2.

### EV uptake and Elisa assays

The EV samples were quantified and added into THP-1 cell culture. Then the conditioned media was harvested after 24 h EV treatment. Next, the supernatant was collected after centrifugation at 400 g for 5 min following by 2000 g for 20 min. The supernatant was used to detect MMP9 level by using human MMP-9 quantitative kits (Quantikine®, R&D Systems) according to the manufacturer's instructions, while it was used to measure IL-6 level by using human IL-6 specific ELISA kits (Human IL6 SuperSet ELISA Kit, ELISAGenie) according to the manufacturer's protocol.

#### Statistical analysis

Plots represent the mean and standard error of the mean (SEM) for at least three independent replicates. Two experimental groups were compared using Student's *t*-test (Ratio-paired, two tails) and *p*value < 0.05 was considered statistically significant (0.01 < *p* value (*) <0.05, 0.001< *p* value (**) < 0.01, *p* value (***) < 0.001). All statistical analyses were performed using GraphPad Prism to calculate statistical significance.

## Results

### Blood cancer EVs induce pro-inflammatory MMP-9 and IL-6 secretion from monocytic cells

Extracellular Vesicle (EV) -cell communication is mediated by either direct interaction with receptors on the cell surface or by delivering a cargo of nucleic acids and proteins that alter the phenotype of the recipient cell [24]. EVs from solid tumours such as breast and pancreatic cancer have been previously shown to increase the secretion of several pro-inflammatory factors including MMP9 and IL-6 from THP-1 monocytic cells [25]. Upregulation of MMP9 and IL-6 promotes tumour invasion [26], proliferation, apoptosis and metastasis [27,28].

**Fig. 2:**
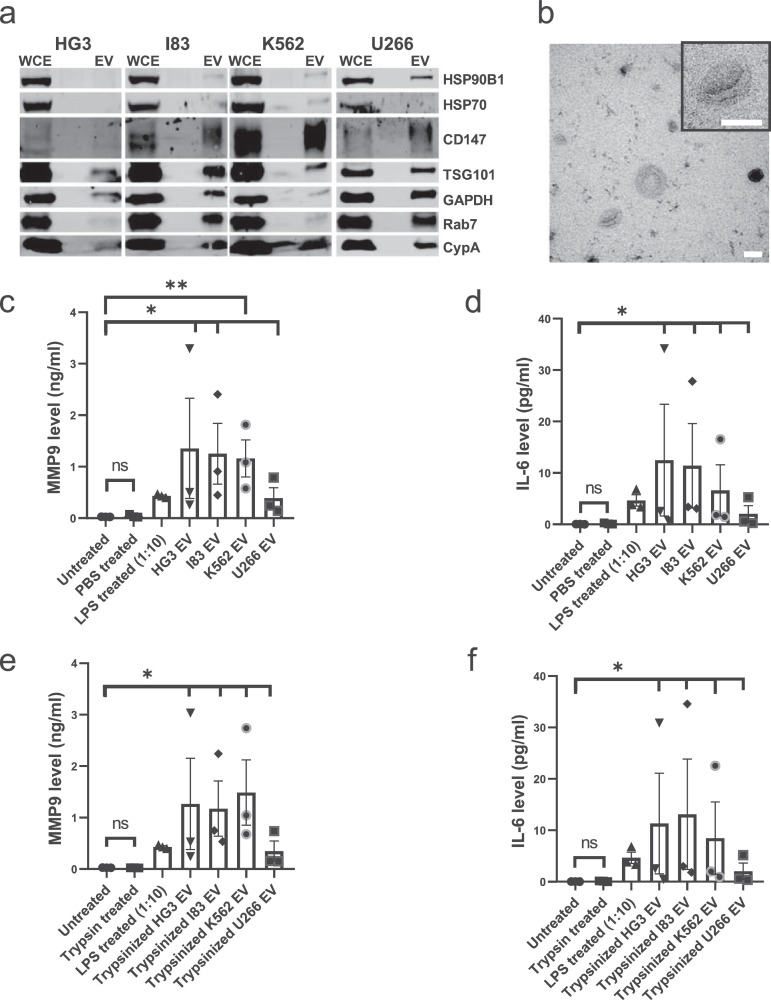
Blood cancer EVs contain CypA and contribute to a pro-inflammatory response from immune cells. (a) CypA is ubiquitously abundant in high-purity EVs from HG3, I83, K562, U266 blood cancer cell lines (1 × 10^6^/ml) isolated by density gradient ultracentrifugation based on various EV associated markers. (b) Representative negative staining transmission electron microscopy images of K562 EVs. Ten microliters of each EV sample was loaded on the grids for TEM imaging. Scale bars= 100 nm. MMP9 and IL-6 are secreted from THP-1 cells upon stimulation with high-purity untrypsinized (c, d) or trypsinized (e, f) EVs from the cancer cell lines. 50 µl of high-purity EVs was isolated from 40 ml conditioned media of equal cell density of HG3, I83, K562, U266 (1 × 10^6^/ml) by density gradient ultracentrifugation. Conditioned media from THP-1 cells (5 × 10^5^/ml) was collected after 24 h stimulation by adding equal volume of high-purity EVs from these blood cancer cell lines. The level of MMP9 and IL-6 were measured in conditioned media after centrifuging at 400 g/ 5 min and 2000 g/ 20 min for removal of cells and cell debris respectively. 50 µl of trypsinized high-purity EVs were obtained by the treatment with 20 µg/ml trypsin at 37 °C for 30 min. (c-f) All bars show the mean ± SEM of three independent experiments. WCE=Whole Cell Extracts, EV= high-purity Extracellular Vesicles.

To date, the effect of blood cancer EVs on MMP-9 and IL-6 secretion remains unknown. In this study, to examine if blood cancer EVs could also induce secretion of MMP9 and IL-6 from monocytes, EVs were isolated from a variety of malignant haematopoietic cells including leukaemia (HG3, I83, K562) and myeloma (U266) by differential centrifugation and iodixanol density flotation gradient (Fig 1). In accordance with the minimal experimental requirements for definition of extracellular vesicles guidelines [29], isolation of EVs was confirmed by the presence of EV proteins and non-vesicular proteins markers (Fig 2a) and transmission electron microscopy (TEM) analysis (Fig 2b, 1S).

To examine if blood cancer EVs could increase MMP9 and IL-6 secretion in monocytes, THP-1 cells were treated with either HG3, I83, K562 and U266 EVs for 24 h and the levels of MMP9 and IL-6 in the THP-1 conditioned media was determined. Results reveal that MMP9 (Fig 2c) and IL-6 (Fig 2d) secretion was significantly upregulated in response to all cancer EVs, relative to untreated cells, while the amount of MMP9 or IL-6 induced was dependent on the cancer cell secreting the EVs. Cells were treated with PBS and LPS as negative and positive controls, respectively.

**Fig. 3:**
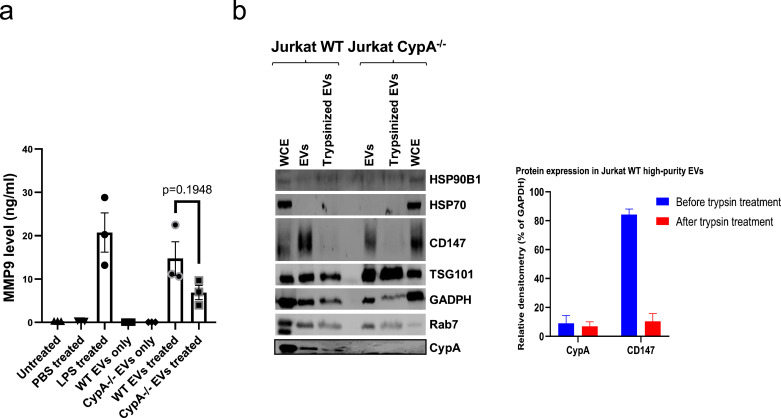
CypA is partially responsible for EV-stimulated MMP9 secretion and is an intravesicular EV protein. (a) MMP9 is secreted from THP-1 cells upon stimulation with Jurkat WT and CypA-/- high-purity EVs. High-purity EVs were isolated from equal volume of conditioned media of equal cell density of Jurkat WT and Jurkat CypA-/- cells (1 × 10^6^/ml) by density gradient ultracentrifugation. Conditioned media from THP-1 cells (5 × 10^5^/ml) was collected after 24 h stimulation with equal volume of high-purity EVs from both cell lines. The level of MMP9 was measured as before. The bars show the mean ± SEM of three independent experiments. (b) CypA is distributed in Jurkat high-purity EVs with/without trypsin treatment. Jurkat WT and CypA-/- EVs were treated with/without 20 µg/ml trypsin for trypsin-sensitive EV surface protein digestion and analysed by western blotting. Densitometry analysis for CypA and CD147 antibodies in Jurkat WT was performed. WCE=Whole Cell Extracts, EV= high-purity Extracellular Vesicles.

**Fig. 4:**
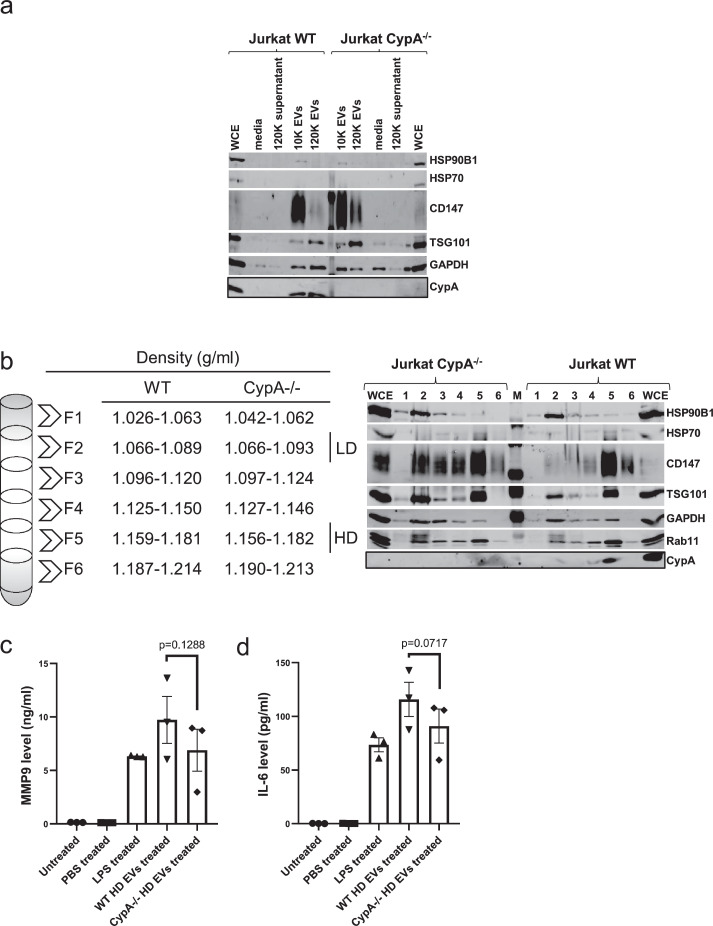
High density EVs contain CypA and stimulate MMP9 and IL-6 secretion. (a) CypA is equally distributed in Jurkat 10 K and 120 K EVs. 10 K and 120 K EV pellets from equal volume of conditioned media of equal cell density of Jurkat WT and Jurkat CypA-/- cells (1 × 10^6^/ml) were isolated by differential ultracentrifugation at 10,000 g and 120,000 g respectively. Both pellets were resuspended in PBS and ultracentrifugated at 120,000 g to remove soluble proteins . 10 K and 120 K EV samples were analysed by western blotting. (b) Jurkat EV subpopulations within a specific density range were fractionated and therein CypA is distributed in high density (HD) EVs and absent from low density (LD) subpopulations. Crude EVs from equal volume of conditioned media of equal cell density of Jurkat WT and Jurkat CypA-/- cells (1 × 10^6^/ml) were enriched by ultracentrifugation at 120,000 g and were fractionated by density gradient ultracentrifugation into different fractions (~1.03-~1.21 g/ml) . Low density EVs were mainly distributed in ~1.06-~1.09 g/ml, while high density EVs were mainly distributed in ~1.15-~1.18 g/ml. Each fraction was resuspended in PBS and ultracentrifuged at 120,000 g and analysed by western blotting. (c, d) MMP9 and IL-6 are secreted from THP-1 cells upon stimulation with Jurkat WT and CypA-/- HD EVs. Conditioned media from THP-1 cells (2.5 × 10^5^/ml) was collected after 24 h stimulation by adding equal volume of Jurkat WT and Jurkat CypA-/- HD EVs. The level of MMP9 and IL-6 were measured as before. (a, c, d) All bars show the mean ± SEM of three independent experiments. WCE=Whole Cell Extracts, EV= high-purity Extracellular Vesicles.

**Fig. 5:**
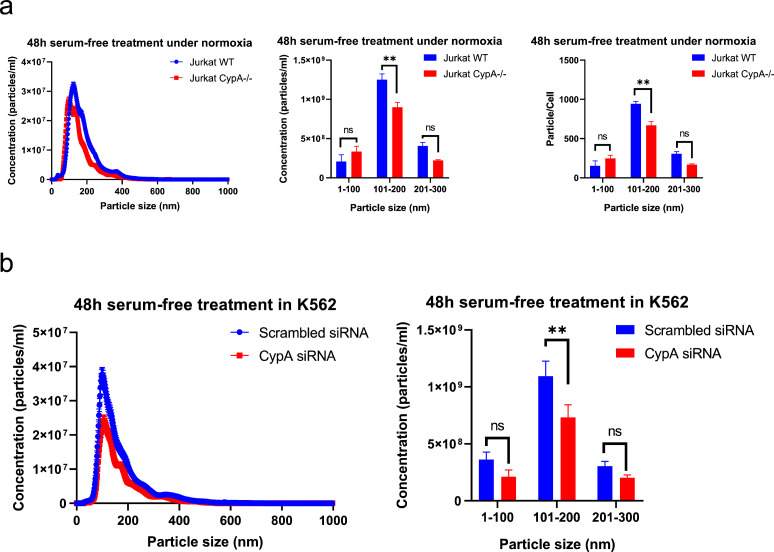
Loss of CypA expression significantly reduces the secretion of EVs ranging from 100 nm to 200 nm. (a) Homozygous loss of CypA reduces EV secretion in Jurkat cells. EV secretion was measured by detecting particles in conditioned media of Jurkat WT and CypA-/- cells (1 × 10^6^/ml) after 48 h serum-free treatment and centrifuging at 400 g/ 5 min and 2000 g/ 20 min for removal of cells and cell debris respectively. Equal volume of conditioned media from both cell lines were equally diluted and measured by nanoparticle tracking analysis (NTA). The line graph represents particle concentration and size distribution ranging from 0 to 1000 nm (Blue= conditioned media from Jurkat WT cells, Red= conditioned media from Jurkat CypA-/- cells). The bar charts represent particle concentration within different size ranges (0–100 nm, 101–200 nm, 201–300 nm) in conditioned media, normalised to particles/ml and particles/cell. (b) Knockdown of CypA reduces EV secretion in K562 cells. EV secretion was measured by detecting particles in conditioned media of K562 cells  (1 × 10^6^/ml) following CypA siRNA knockdown and after 48 h serum-free treatment. Media was centrifuged at 400 g/ 3 min and 2000 g/ 20 min for removal of cells and cell debris respectively. Equal volume of conditioned media from K562 cells under two conditions were equally diluted and measured by NTA. The line graph represents particle concentration and size distribution from 0 to 1000 nm (Blue= conditioned media from K562 cells with scrambled siRNAs, Red= conditioned media from K562 cells with pooled CypA siRNAs). The bar chart represents particle concentration within different size ranges in conditioned media. (a, b) All bars show the mean ± SEM of three independent experiments (0.001< *p* value (**) < 0.01).

EVs have been shown to induce signalling by interacting with proteins on the recipient cell surface or after they are internalized by cells through receptor-mediated endocytosis (clathrin-mediated, lipid raft-mediated and caveolin-dependent endocytosis) or by receptor-independent processes such as macropinocytosis, membrane fusion and phagocytosis [24]. To determine if THP-1 cells can take up blood cancer EVs, THP-1 cells were incubated with increasing Jurkat EVs labelled with CFDA. EV uptake was confirmed by the time-dependent and dose-dependent increase in CFDA fluorescence observed from the THP-1 cells (Fig 2S). Several studies report that trypsin or proteinase K treatment of EVs degrades EV surface proteins, however, it does not penetrate the lipid bilayer [30], [31], [32]. In this study, the concentration of trypsin was optimised for EV treatment (Fig 3S), and TEM images of blood cancer EVs before and after treatment with high concentration of trypsin (20 µg/ml) (Fig 4S) confirm that EVs are intact after treatment. Moreover, the EV surface trypsin-sensitive protein CD147 was cleaved using high concentration of trypsin, while the CFDA fluorescence was sustained after high concentration of trypsin treatment of the EVs (Fig 5S). Collectively these data demonstrate that the EV uptake by THP-1 cells was unaffected by protease treatment at concentrations sufficient to remove surface proteins, suggesting that the process of EV internalisation is receptor-independent.

In order to determine whether the induction of MMP9 and IL-6 was due to protein interactions at the recipient cell surface or the direct delivery of EV cargo, the EV surface proteins were cleaved by treatment with high concentration of trypsin (20 µg/ml) prior to incubation with the THP-1 cells. Results reveals that MMP9 (Fig 2e) and IL-6 (Fig 2f) secretion was unchanged following EV trypsinization (Fig 2c, d vs Fig 2e, f) and suggests that the delivery of the EV cargo is responsible for increasing MMP9 and IL-6 levels.

### CypA is partially responsible for EV-stimulated MMP9 secretion

**Fig. 6:**
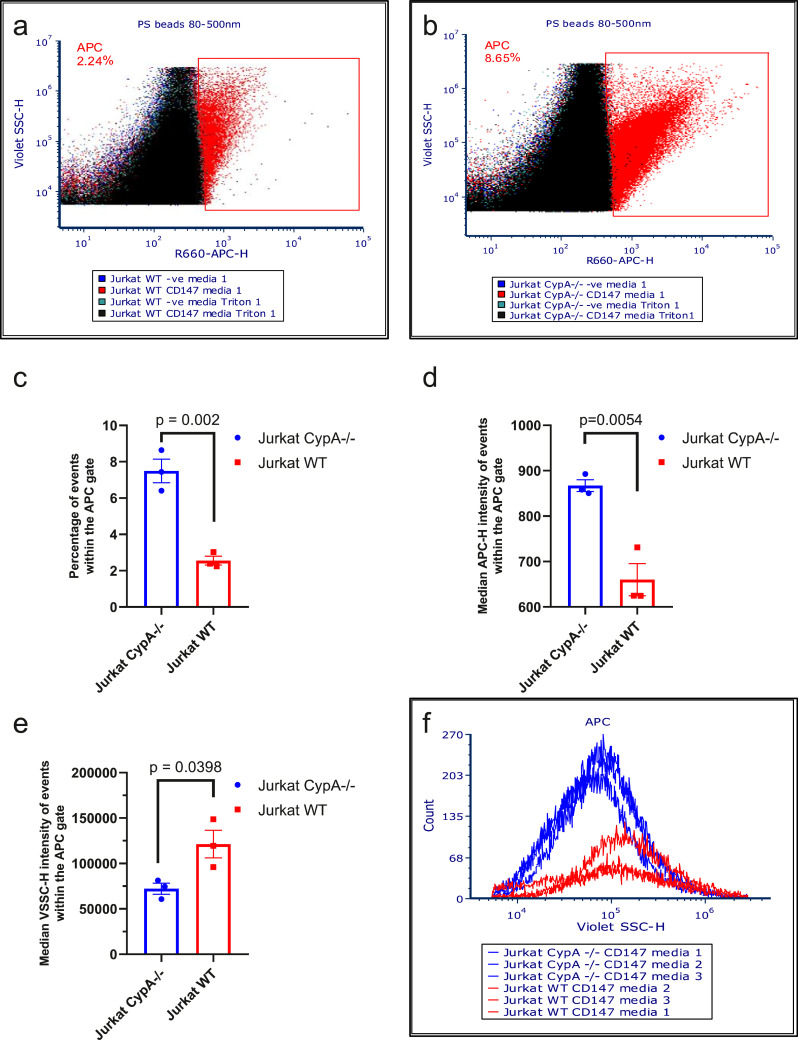
CypA knockout increases surface CD147 expression and reduces the size of CD147+ EVs in conditioned media. Surface CD147 expression was measured on EVs in conditioned media from Jurkat WT (a) and CypA-/- (b) cells (1 × 10^6^/ml) after 48 h serum-free treatment and centrifuging at 400 g/ 5 min and 2000 g/ 20 min for removal of cells and cell debris respectively. Conditioned media from both cell lines were equally diluted to 5000 events/s before incubation with 0.1μl anti-CD147-APC for 30 min on ice and/or 0.1% triton for an additional 30 min. Dot-plots represent an overlay of events within the PS beads 80–500 nm gate (Representative gating strategy detailed in fig 9S) from unstained (-ve), CD147 stained and Triton lysed EV samples. An APC gate was setup using the anti-CD147-APC antibody in PBS in order to identify CD147+ events (fig 9S F). The percentages in the dot plots indicate the number of CD147 positive EVs amongst the total EVs. The events from conditioned media of Jurkat WT and CypA-/- cells from 3 independent experiments within the APC gate were plotted on bar graphs to represent (c) the average percentage of events that are CD147-APC+, (d) the average median APC—H intensity of events within the APC gate, (e) the average median Violet SSC—H intensity of events within the APC gate. (f) The events from conditioned media of Jurkat WT and CypA-/- cells from 3 independent experiments within the APC gate were plotted overlaid on Violet SSC—H histograms to compare the relationship between event number and particle size for each experiment. Error bars represent SEM of 3 independent experiments.

CypA is a highly abundant cellular protein and in this study we show that it is present in cells and EVs from leukaemia and myeloma cells (Fig 2a). To examine if CypA is responsible for the EV-induced pro-inflammatory signals, MMP9 secretion was measured in THP-1 after 24 h treatment with EVs from an equal number of Jurkat cells (WT) or Jurkat cells with homozygous knockout of CypA (CypA-/-) cultured for 48 h in serum-free media. Cell number and viability were confirmed to be similar in the two cell lines (Fig 6S). MMP9 secretion was significantly increased from THP1 cells in response to Jurkat WT and CypA-/- EVs (Fig 3a), however, the extent of MMP9 secretion induced by CypA-/- EVs was reduced relative to WT EVs, and although this was not statistically significant (*p* value = 0.1948), it suggests that CypA contributes in part to MMP9 signalling. Whether this is due to intracellular CypA or EV-associated CypA, or both, remains to be determined.

### CypA is an intravesicular EV protein

To determine whether CypA was associated with the EV surface or packaged inside the EV core, characterisation of CypA in EVs was performed using western blot analysis of trypsinized and untrypsinized EVs. Results reveal that CypA is present in the EVs before and after trypsinization, whereas the EV surface protein CD147 was degraded following trypsinization of the EVs, indicating that CypA is a core EV protein and not expressed on the EV surface (Fig 3b).

### High-density EVs that contain CypA stimulate pro-inflammatory MMP9 and IL-6 secretion

Previous studies report that EV cargo differs in subpopulations of EVs with different sizes and densities [33], [34], [35]. In order to investigate whether CypA is associated with a subpopulation of EVs, small and large EVs isolated from Jurkat cells (WT and CypA-/-) were fractionated into a 10 K and 120 K EV fractions respectively (Fig 4a) and probed for CypA. CypA was detected in small and large EVs, whereas CD147 and HSP90B1 are mainly expressed in 10 K EVs, and TSG101 was more prominent in 120 K EVs. Thus, it is not possible to assign CypA to a distinct EV subgroup based on size. Possible reasons are that it is present in both small and large EVs, or that it is present in medium sized EVs that cannot be easily separated using available technology. Furthermore, it was found that loss of CypA in EVs did not significantly alter the distribution of the EV markers examined.

To determine whether CypA varies in EV subpopulations based on density, EVs were separated into six fractions by differential centrifugation and iodixanol density flotation gradient (36%, 24%, 20%, 10%). EVs were distributed across all fractions with fraction 2 and fraction 5 having a higher proportion of EV markers CD147 and TSG101(Fig 4b). Results reveal that CypA is found in 1.15–1.18 g/ml fraction (high density EVs, HD). Interestingly, it was also observed that these high density EVs have higher levels of HSP70 and Rab11, and lower levels of HSP90B1 and GAPDH relative to 1.06–1.09 g/ml fraction (low density EVs, LD) suggesting that CypA contributes to alteration of EV protein signature.

Considering heterogeneity of EV cargos in EV subpopulations based on density, we investigated whether the loss of CypA from high-density EVs was responsible for the decreased MMP9 secretion from monocytes. THP-1 cells were treated for 24 h with high density EVs purified by iodixanol density flotation from Jurkat WT and CypA-/- cells, and MMP9 levels were measured. Results demonstrate that MMP9 secretion from THP-1 cells is increased in response to the high density EVs from Jurkat WT cells, and this is attenuated following incubation with high density EVs from CypA-/- cells (Fig 4c), which is consistent with data reported earlier with unfractionated EVs from both cell types (Fig 3a). Furthermore, it was found that in addition to MMP9, high density EVs from Jurkat WT cells significantly increase secretion of IL-6 from monocytes, which is attenuated following loss of CypA in Jurkat CypA-/- cells (Fig 4d).

**Fig. 7:**
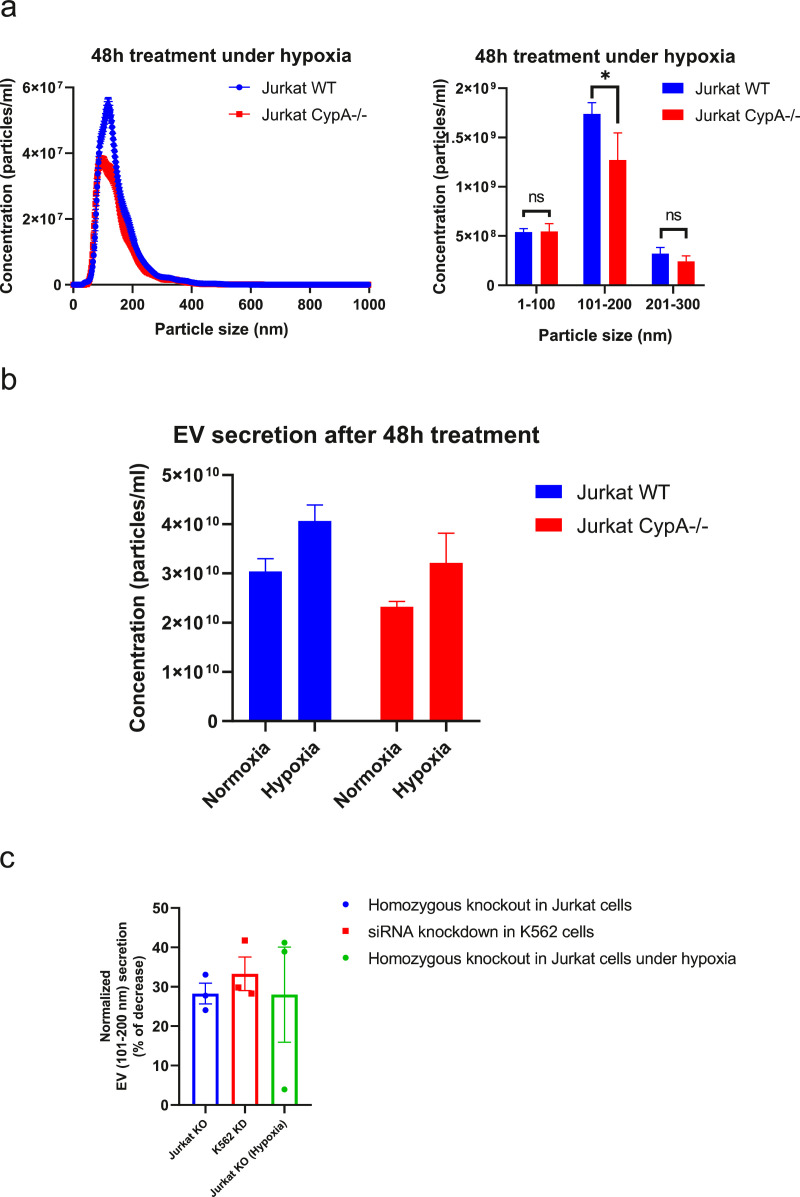
Loss of CypA expression reduces the release of EVs under hypoxia. (a) Homozygous loss of CypA reduces EV secretion in Jurkat cells under hypoxic conditions. EV secretion was measured by detecting particles in conditioned media of Jurkat WT and CypA-/- cells (1 × 10^6^/ml) after a low concentration of FBS treatment under hypoxia (1% O_2_) for 48 h and centrifuging at 400 g/ 3 min and 2000 g/ 20 min for removal of cells and cell debris respectively. Equal volume of conditioned media from both cell lines were equally diluted and measured by NTA. The line graph represents particle concentration and size distribution from 0 to 1000 nm (Blue= conditioned media from Jurkat WT cells, Red= conditioned media from Jurkat CypA-/- cells). The bar chart represents particle concentration within different size ranges in conditioned media. (b) The total EV concentration (0–1000 nm) after 48 h treatment was calculated under normoxic and hypoxic conditions. (c) The concentration of particles in 101–200 nm range secreted into conditioned media was decreased under three conditions as shown by% of decrease. (a-c) All bars show the mean ± SEM of three independent experiments (0.001< *p* value (**) < 0.01).

In addition to EV normalisation based on equal cell number and volume of conditioned media, as presented in Fig 4, two other EV quantification methods were used to normalise EVs including number from nanoparticle tracking analysis (NTA) and protein concentration from bicinchoninic acid (BCA) protein assay prior to incubation with THP-1 cells and pro-inflammatory signal measurement. Results from experiments using the alternative normalisation methods showed MMP9 secretion from THP-1 cells was marginally increased in response to Jurkat WT EVs relative to Jurkat CypA-/- EVs (Fig 7S). Overall, results from three normalisation methods showed differential secretion of pro-inflammatory signals from the recipient cells, despite a compensatory increase in other EV components in CypA-/- fraction, suggesting that loss of CypA in Jurkat cells alters the population of EVs released and as a result the pro-inflammatory response from monocytes. The difference in extent of signal detected using different normalisation methods prompted us to investigate if it was due to alteration in the EV profile secreted in the presence and absence of CypA from cells.

### CypA regulates release of EVs from blood cancer cells

**Fig. 8:**
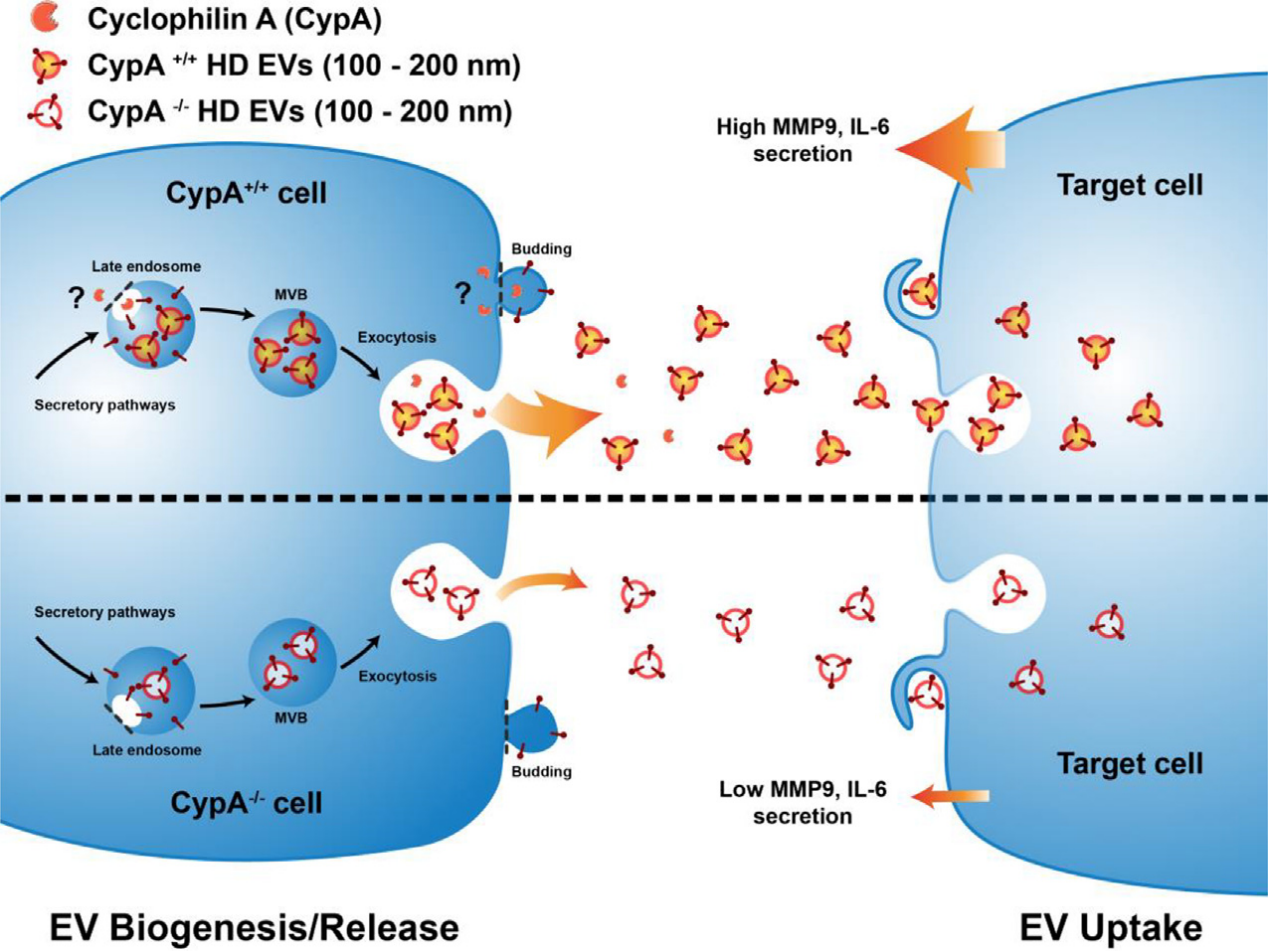
Proposed model of a role for CypA in cancer EV biogenesis and function. CypA is ubiquitously distributed in blood cancer cells and is associated with high density (HD) EVs in the range of 100 to 200 nm secreted by Jurkat cells.  Loss of CypA expression reduces the quantity and alters the profile of the secreted EV population, which attenuates the inflammatory response from recipient immune cells.

Results from this study so far demonstrate that blood cancer-derived EVs significantly enhance MMP9 and IL-6 secretion from monocytes, which is attenuated by deletion of CypA. Intracellular CypA has a well-established role in viral budding [36,37] and membrane abscission during cytokinesis [20], processes which are topologically similar to EV biogenesis, and supports the rationale that the reduction of EV-induced MMP9 and IL-6 secretion following loss of CypA was due to alterations in EV production and secretion. To test this, conditioned media was collected from an equal number of Jurkat WT and CypA-/- cells following 48 h culture in serum-free media and EV number and size distribution was measured. NTA data revealed that loss of CypA expression in Jurkat cells result in a 23.5% reduction in EV number secreted (0–1000 nm) (Fig 5a), with EVs in size range of 100–200 nm being significantly reduced. Furthermore, EV number (0–1000 nm) was reduced by 35% in K562 cells following siRNA-mediated knockdown of CypA with EVs in size range of 100–200 nm being significantly reduced (Fig 5b). siRNA knockdown and cell viability after 48 h serum-free media treatment was confirmed and demonstrates that the reduction in EVs released was not due to cell death following deletion of CypA (Fig 8S). Thus, this data reveals a novel role for CypA in the production of EVs of 100–200 nm in size from blood cancer cells.

To determine if the reduction in EV number in the CypA-/- cells resulted in a change in profile of the EV population, expression of the EV marker CD147 (EMMPRIN/BSG) was examined by flow cytometry (29). Conditioned media was collected from an equal number of Jurkat WT and CypA-/- cells following 48 h culture in serum-free media and the conditioned media was diluted with filtered PBS to achieve an equal concentration of particles that would not induce swarm effects when recorded (5000 events/sec). The EV samples were stained with anti-CD147-APC and recorded for 2 min on the Cytoflex. Events were gated to remove EV aggregates (singlet gate), and gated based on particle size (gates set against 80 nm and 500 nm polystyrene beads). An APC gate was set to exclude the background signal of the anti-CD147 antibody in PBS (Fig 9S). CD147-APC^+^ particles were detected in both WT and CypA-/- samples (Fig 6a, b), with a higher percentage of CD147-APC^+^ particles present in CypA-/- samples (Fig 6c, Table S1). The CD147-APC^+^ particles present in CypA-/- samples had a higher median APC—H intensity (Fig 6d) and a lower VSCC—H than WT samples (Fig 6e). The lower median VSCC—H is due to an increase in smaller CD147-APC^+^ particles in CypA-/- samples relative to the WT samples (Fig 6f). EV samples were lysed with 0.1% triton and recorded on the Cytoflex in order to confirm that the CD147-APC^+^ particles were EVs (Fig 6a, b). The change in CD147 expression was also confirmed in high-density EVs (Fig 10S) and by western blot analysis (Fig 11S).

### CypA regulates release of blood cancer derived EVs under hypoxia

CypA is over-expressed in various cancer types in response to hypoxia [38,39], and hypoxia has been previously shown to increase EV secretion [40], [41], [42], [43]. Based on that, we examined the impact of CypA expression on EV secretion under hypoxic conditions. To maintain cell viability under hypoxic conditions, cells were incubated in low concentration of FBS (3%) (Fig 12S) for 48 h and EV number and size distribution was measured by NTA. Results reveal that EV secretion is increased by 1.4-fold by both Jurkat WT and CypA-/- cells in response to hypoxia (Fig 7b), which is consistent with literature reports that hypoxia increases EV secretion. Moreover, it was found that Jurkat CypA-/- cells have a significantly reduced secretion of EVs in the size range of 100–200 nm relative to WT cells under hypoxic conditions (Fig 7a) which is consistent with data reported under normoxic conditions (Fig 5a). Collectively these data reveal a novel function for CypA in the biogenesis and/or release of EVs (100–200 nm in diameter) from cancer cells under normoxic and hypoxic conditions (Fig 7c).

## Discussion

Tumour-derived EVs are vital regulators of intercellular communication between tumour cells and normal cells within the tumour microenvironment for pre-metastatic niche initiation via the establishment of a pro-inflammatory milieu [44], [45], [46], [47], [48], [49], [50], [51]. Indeed, EVs can directly interact with different cell types including immune cells where they induce an inflammatory response. For example, it has been shown that the secretion of pro-inflammatory factors including MMP-9, IL-6 and TGF-β1 from monocytes was upregulated in response to cancer-derived EV stimulation [25]. Similarly, in this study we report that secretion of MMP9 and IL-6 were significantly increased from monocytes upon stimulation with EVs from several blood cancer cell lines. The extent of pro-inflammatory signals from monocytes differed following stimulation with EVs from different blood cancer, which suggests that the potential to activate pro-inflammatory signals in the tumour microenvironment may be cancer-type specific, and dependent on EV composition and/or uptake.

Previous evidence suggests that EVs are internalized into recipient cells by either receptor-dependent or -independent mechanisms that vary based on cell type. Many studies have demonstrated that EV proteins can interact with membrane receptors on recipient cells, which mediate uptake into the cells [52,53]; this is supported by the significant reduction in EV uptake by ovarian cancer cells following proteinase K treatment of EVs [54]. Another mechanism for EV uptake is via direct fusion of the EV membrane with the cell plasma membrane [55]. One study reported that trypsin treatment of EVs did not damage the EV structure and there are no significant differences in uptake between the trypsin-treated and the non-treated EVs, demonstrating that uptake is not determined by the surface protein [56]. In this study, protease treatment of EVs led to degradation of EV surface proteins, thereby preventing protein-protein interactions at the recipient monocyte cell surface, without compromising EV uptake. Moreover, MMP9 and IL-6 secretion was unaffected by trypsin treatment, which suggests that the increased secretion is mediated by EV cargo delivery to monocytes and is not mediated through cell surface signalling.

Previous studies demonstrate that CypA is upregulated in various tumour cells and is a key determinant of cancer transformation, metastasis, and chemoresistance [16]. Overexpressed CypA, which acts as a molecular chaperone for other cellular proteins, may have cytoprotective effects during tumorigenesis [57], while CypA has antioxidant effects through its *cis-trans* isomerase activity in cancer cells [58]. In addition, according to EVpedia [59] and Vesiclepedia [60], CypA is detected in EVs from various cancer cell lines. Consistent with that, we demonstrate that CypA is present in EVs from a variety of blood cancer cells. However, the function of CypA in cancer-derived EVs has not been characterised in detail.

EVs are a heterogeneous population of vesicles and general mechanisms of EV biogenesis and secretion are well characterised [61]. EVs in the size range of exosomes (50–200 nm) share some of the same biophysical characteristics in terms of size, density and membrane orientation [62], and recent reports demonstrate the existence of EV subpopulations with distinct molecular and biological properties [35,^63^,64], however to date, these subpopulations remain largely uncharacterised. In this study, EV subpopulations were fractionated into low and high density EVs using high-resolution density gradient, and it was found that CypA is present in high density EVs and is absent from low density EVs. In addition, CypA was detected in EVs following trypsin digestion, suggesting that it is not located on the EV surface, but instead, it is packaged into the EVs during biogenesis. Thus, this study reveals that CypA is a core component of high-density cancer derived EVs. Furthermore, results reveal that other EV associated proteins (TSG101, HSP70 and Rab11) are differentially distributed into low- and high-density subpopulations, which together with CypA, localise within high density EVs, providing evidence for the existence of CypA+/+ and CypA-/- EV subpopulations that carry distinct cargo and highlighting a possible role for CypA in control of EV cargo.

In this study, the role of CypA-positive EVs on monocyte function was investigated using EVs secreted from equal number of the isogenic cell line pair (Jurkat WT and CypA-/-), and proinflammatory MMP9 and IL-6 cytokine signals were measured following EV treatment of monocytes. Results indicate that high density EVs from Jurkat WT cells induce a proinflammatory response from monocytic cells, and high density EVs from Jurkat CypA-/- cells attenuate the response from monocytes, suggesting that CypA modifies the cancer-derived EV pro-inflammatory response. Pro-inflammatory signals including IL-6 and MMP9 secretion are mediated by genetic and epigenetic regulation, therefore it is not surprising that the loss of CypA expression resulted in a modest attenuation of the signal.

The process of EV biogenesis is topologically similar to plasma membrane alterations required for cytokinesis and viral budding, for which CypA has been assigned important roles [21]. More specifically, intracellular CypA plays an important role in membrane abscission of daughter cells during cell division [20]. CypA is also involved in replication of several viruses (HIV-1, HCV, SARS-CoV) [16,65], and has been shown to be required for HIV-1 virus budding from the plasma membrane [36,37]. Furthermore, EV proteins, ALIX and TSG101, that are involved in EV biogenesis [66] are also recruited by viruses during budding from the plasma membrane [67] and are involved in cytokinesis [68], suggesting that common machinery is required for these membrane remodelling processes. This prompted us to investigate whether CypA is involved in EV biogenesis, which would account for the reduction in pro-inflammatory signals detected following loss of CypA.

Consistent with that hypothesis, we demonstrated that in a variety of blood cancer cell lines, loss of CypA expression by gene knockout, or siRNA mediated silencing, significantly reduced the release of EVs in the size range of 100–200 nm which reveals a novel role for CypA in EV biogenesis and/or secretion. The function of CypA in plasma membrane remodelling events suggests a possible role in microvesicle formation or secretion, which remains to the confirmed. The change in secreted EV profile may account for the reduced cytokine secretion by EVs from CypA knockout cells. This change in EV population profile could also explain why normalising the EV treatments by equal EV number was not capable of reversing the attenuation of MMP9 secretion observed in THP-1 cells treated with EVs from CypA-/- cells. This data suggests that not all EV populations can induce MMP9 secretion in THP-1 cells and that CypA-/- cells release a lower percentage of EVs capable of inducing MMP9 secretion. This hypothesis is supported by the increase in the percentage of EVs which are CD147+ released from CypA-/- cells and confirms that loss of CypA changes the profile of the EV population.

Finally, CypA is well established as a hypoxia responsive gene, with HIF1α shown to bind to the CypA promoter to increases CypA expression [39]. Hypoxia also increases EV secretion within the tumour microenvironment [41]. In this study, the role of CypA in EV secretion was investigated under hypoxia. Although EV secretions from Jurkat WT and CypA-/- cells were enhanced under hypoxic conditions compared to normoxic conditions, a significant reduction of EV number in the size range of 100–200 nm was detected following loss of CypA expression. These data demonstrate that CypA also regulates the biogenesis and/or release of EVs (100–200 nm) in response to hypoxia and their release seems to be elevated by CypA presence.

In conclusion, while it is well established that tumour-derived EVs transmit oncogenic signals to normal immune cells for cancer progression [44,69], mechanistic details remain poorly understood. In this study, we describe pro-tumorigenic inflammatory signals mediated by a range of blood cancer derived EVs. Furthermore, we reveal that depletion of CypA significantly reduces the release of high density EVs in the range of 100–200 nm (Fig 8) from blood cancer cells. Overall, this study provides new insight into EV biogenesis and reveals CypA as an attractive target for the reduction of tumour EV secretion within the tumour microenvironment.

## Funding

This work was funded by through a UCD-CSC Scholarship awarded to Wu Yunjie. Funding is also acknowledged from the UCD Wellcome Institutional Strategic Support Fund, which was financed jointly by University College Dublin and the SFI-HRB-Wellcome Biomedical Research Partnership (ref. 204,844/Z/16/Z). Authors also acknowledge support of the University College Dublin EQUIP funding scheme.

## Author contributions

Yunjie Wu: Methodology, Data Curation, Writing- Original draft preparation, Writing- Reviewing and Editing, Funding acquisition

K Brennan: Methodology, Data Curation, Writing- Original draft preparation, Writing- Reviewing and Editing

A Blanco Fernandez: Methodology, Reviewing and Editing

M Mc Gee: Conceptualization, Methodology, Supervision, Writing- Reviewing and Editing, Funding acquisition

## Declaration of Competing Interest

The authors declare that they have no known competing financial interests or personal relationships that could have appeared to influence the work reported in this paper.

## Appendix Supplementary materials

Image, application 1

Image, application 2
