# Supplementary material for: Cyclophilin A regulates secretion of tumour-derived extracellular vesicles
Source: Transl Oncol. 2021 May 10;14(8):101112. doi: 10.1016/j.tranon.2021.101112 (PMC8131927; doi:10.1016/j.tranon.2021.101112)
Supplement: Supplementary file 1 [file mmc1.pptx]

## Slide 1
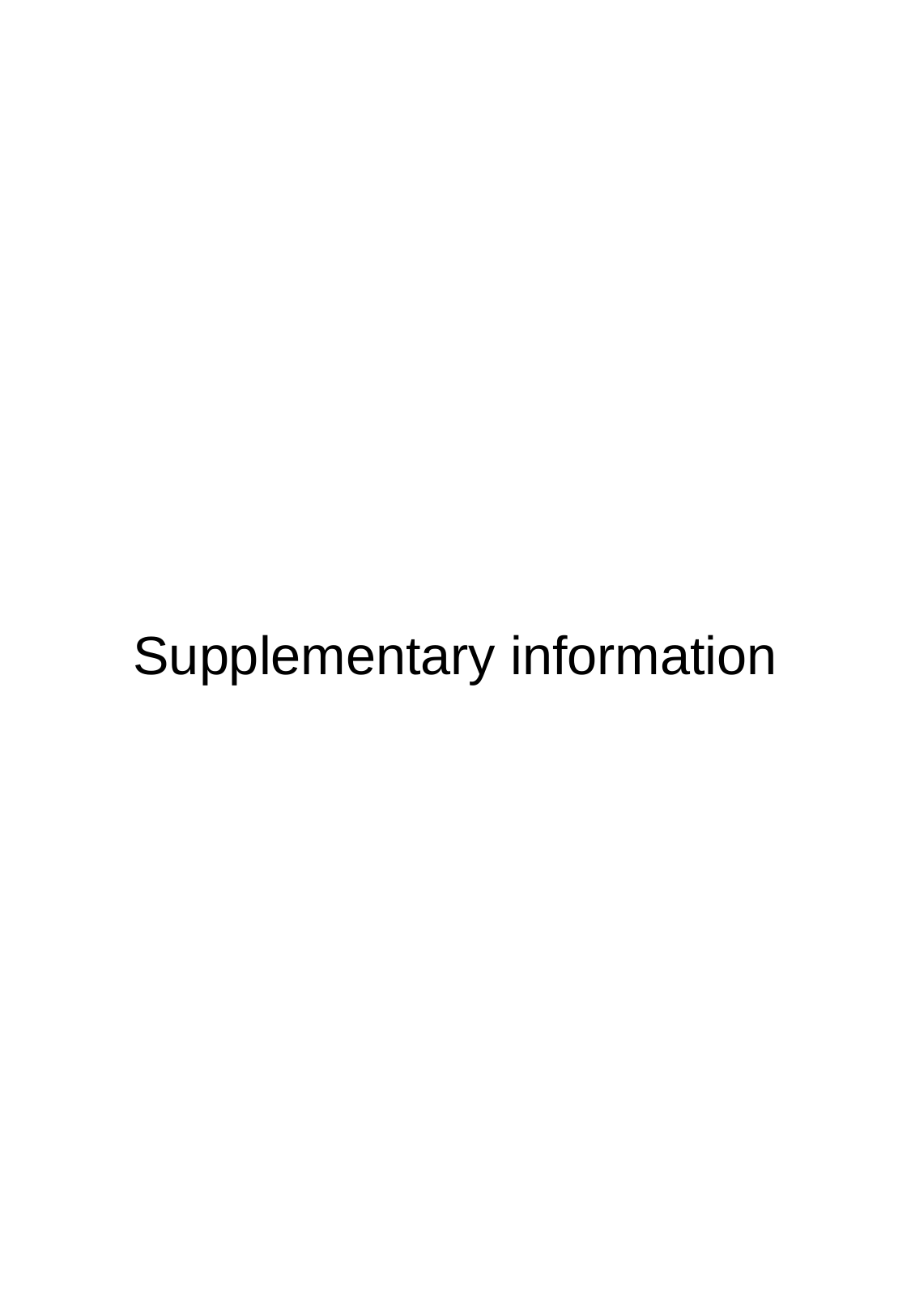

# Supplementary information

## Slide 2
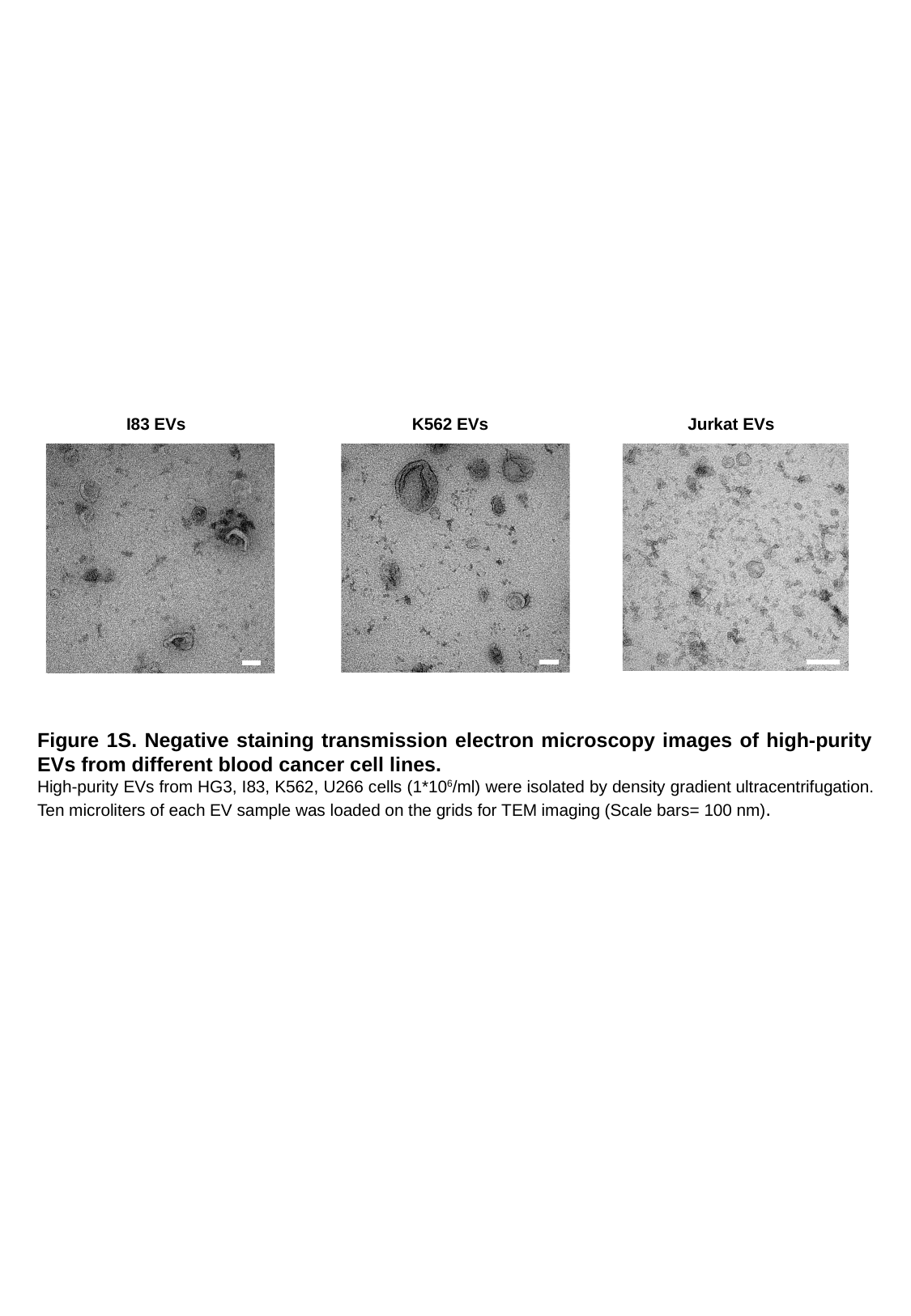

I83 EVs
K562 EVs
Jurkat EVs
Figure 1S. Negative staining transmission electron microscopy images of high-purity EVs from different blood cancer cell lines.
High-purity EVs from HG3, I83, K562, U266 cells (1*106/ml) were isolated by density gradient ultracentrifugation. Ten microliters of each EV sample was loaded on the grids for TEM imaging (Scale bars= 100 nm).

## Slide 3
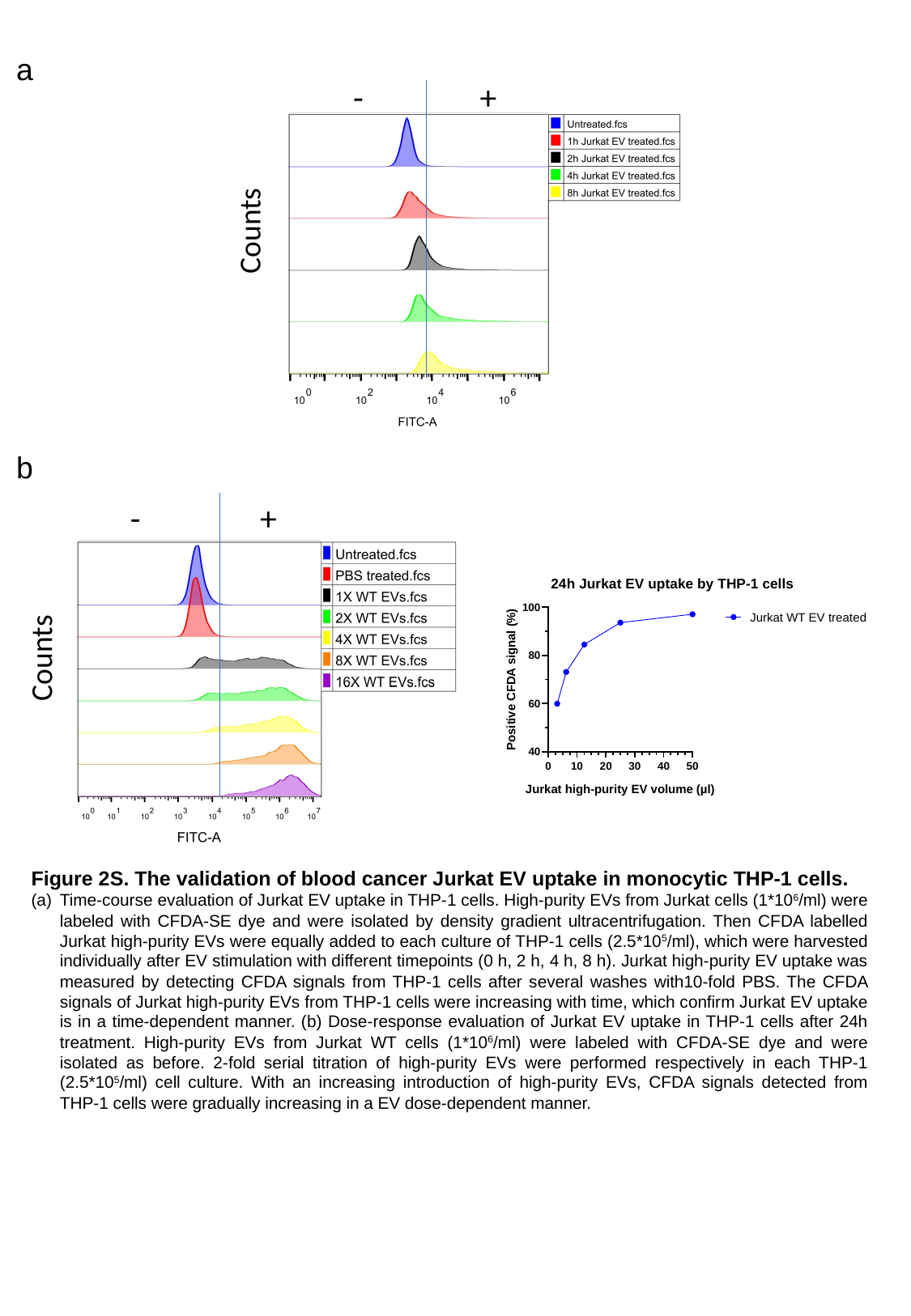

a
-
+
Counts
b
-
+
Counts
Figure 2S. The validation of blood cancer Jurkat EV uptake in monocytic THP-1 cells.
Time-course evaluation of Jurkat EV uptake in THP-1 cells. High-purity EVs from Jurkat cells (1*106/ml) were labeled with CFDA-SE dye and were isolated by density gradient ultracentrifugation. Then CFDA labelled Jurkat high-purity EVs were equally added to each culture of THP-1 cells (2.5*105/ml), which were harvested individually after EV stimulation with different timepoints (0 h, 2 h, 4 h, 8 h). Jurkat high-purity EV uptake was measured by detecting CFDA signals from THP-1 cells after several washes with10-fold PBS. The CFDA signals of Jurkat high-purity EVs from THP-1 cells were increasing with time, which confirm Jurkat EV uptake is in a time-dependent manner. (b) Dose-response evaluation of Jurkat EV uptake in THP-1 cells after 24h treatment. High-purity EVs from Jurkat WT cells (1*106/ml) were labeled with CFDA-SE dye and were isolated as before. 2-fold serial titration of high-purity EVs were performed respectively in each THP-1 (2.5*105/ml) cell culture. With an increasing introduction of high-purity EVs, CFDA signals detected from THP-1 cells were gradually increasing in a EV dose-dependent manner.

## Slide 4
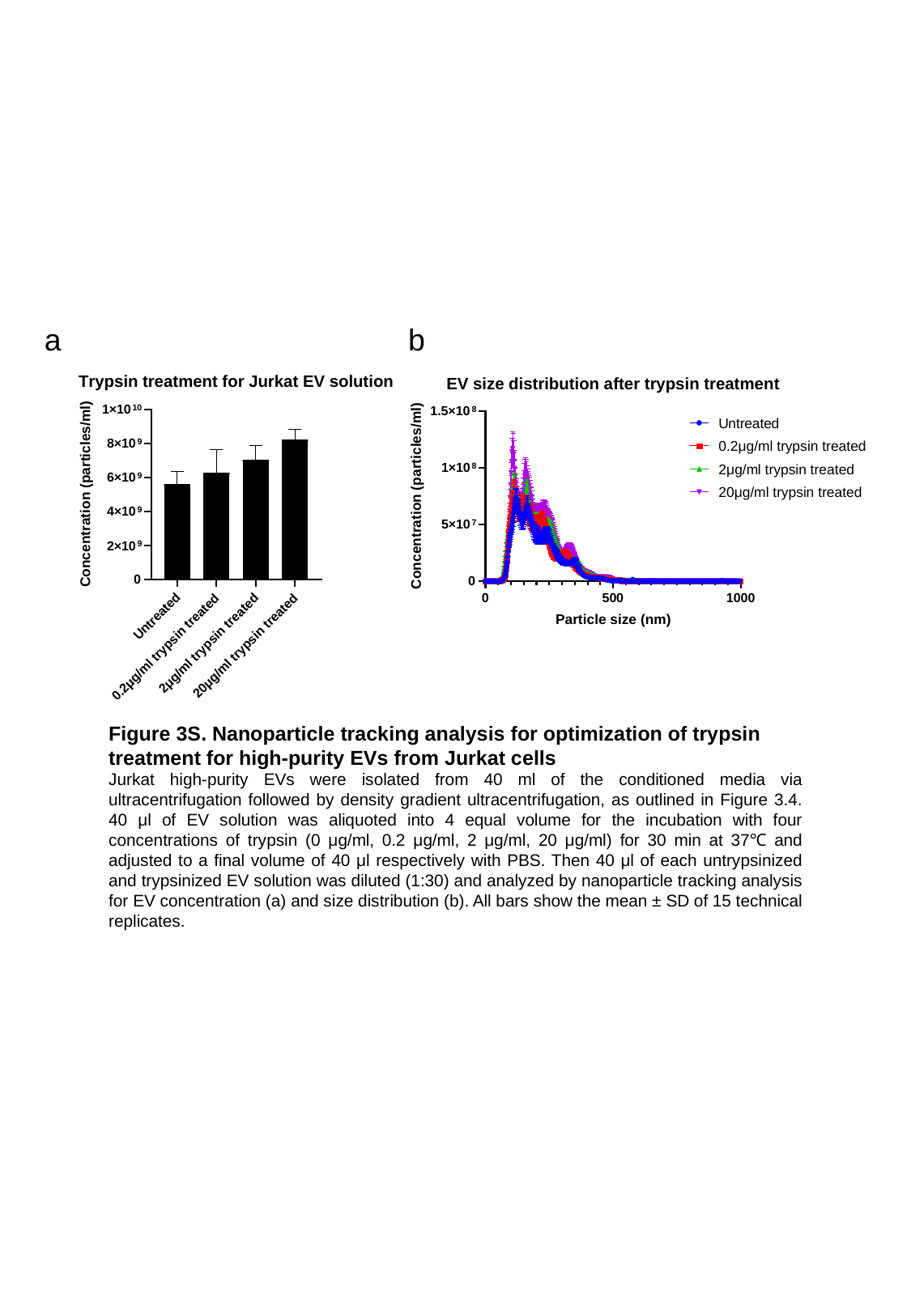

a
b
Figure 3S. Nanoparticle tracking analysis for optimization of trypsin treatment for high-purity EVs from Jurkat cells
Jurkat high-purity EVs were isolated from 40 ml of the conditioned media via ultracentrifugation followed by density gradient ultracentrifugation, as outlined in Figure 3.4. 40 μl of EV solution was aliquoted into 4 equal volume for the incubation with four concentrations of trypsin (0 μg/ml, 0.2 μg/ml, 2 μg/ml, 20 μg/ml) for 30 min at 37℃ and adjusted to a final volume of 40 μl respectively with PBS. Then 40 μl of each untrypsinized and trypsinized EV solution was diluted (1:30) and analyzed by nanoparticle tracking analysis for EV concentration (a) and size distribution (b). All bars show the mean ± SD of 15 technical replicates.

## Slide 5
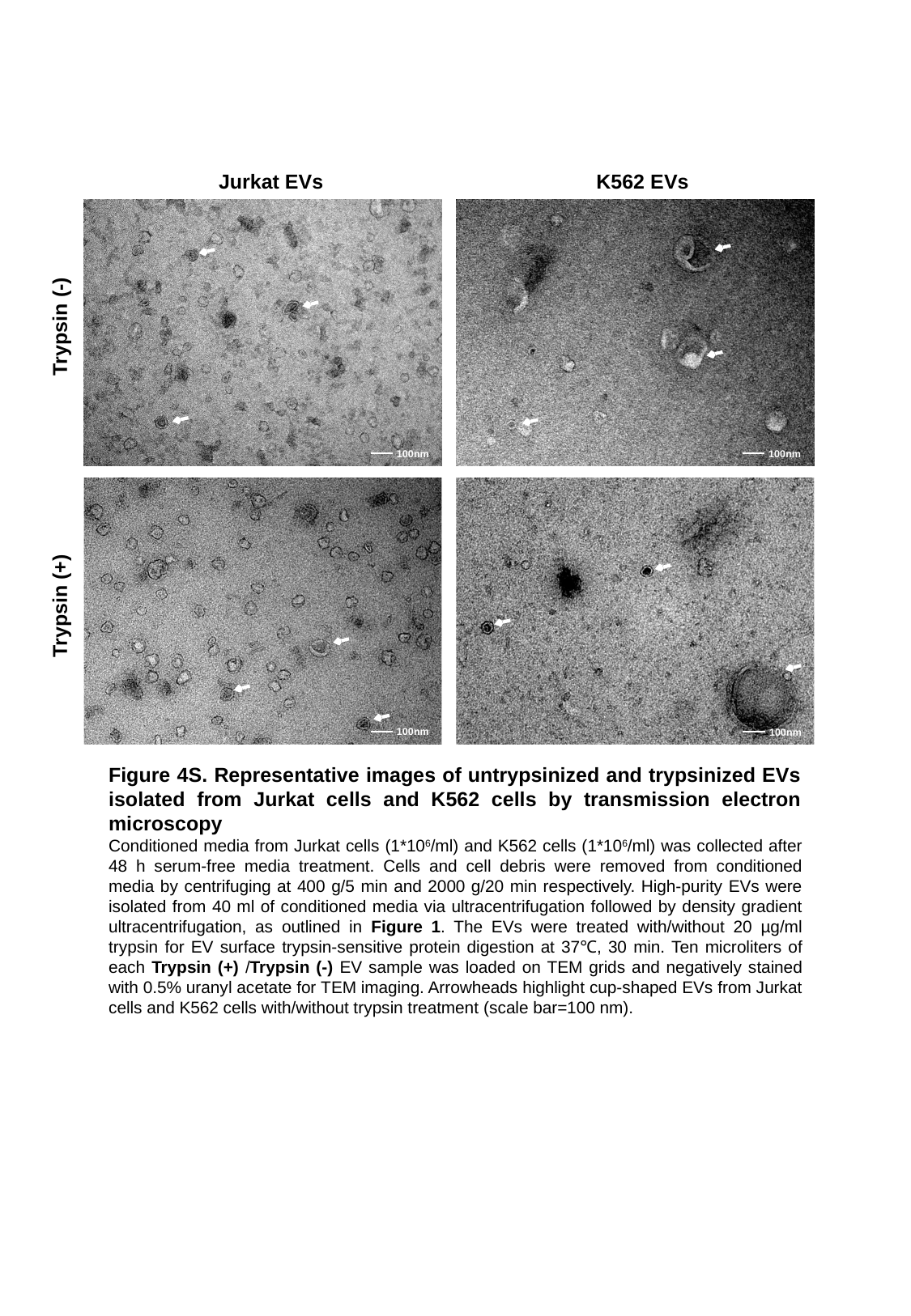

K562 EVs
Jurkat EVs
100nm
100nm
Trypsin (-)
100nm
100nm
Trypsin (+)
Figure 4S. Representative images of untrypsinized and trypsinized EVs isolated from Jurkat cells and K562 cells by transmission electron microscopy
Conditioned media from Jurkat cells (1*106/ml) and K562 cells (1*106/ml) was collected after 48 h serum-free media treatment. Cells and cell debris were removed from conditioned media by centrifuging at 400 g/5 min and 2000 g/20 min respectively. High-purity EVs were isolated from 40 ml of conditioned media via ultracentrifugation followed by density gradient ultracentrifugation, as outlined in Figure 1. The EVs were treated with/without 20 µg/ml trypsin for EV surface trypsin-sensitive protein digestion at 37℃, 30 min. Ten microliters of each Trypsin (+) /Trypsin (-) EV sample was loaded on TEM grids and negatively stained with 0.5% uranyl acetate for TEM imaging. Arrowheads highlight cup-shaped EVs from Jurkat cells and K562 cells with/without trypsin treatment (scale bar=100 nm).

## Slide 6
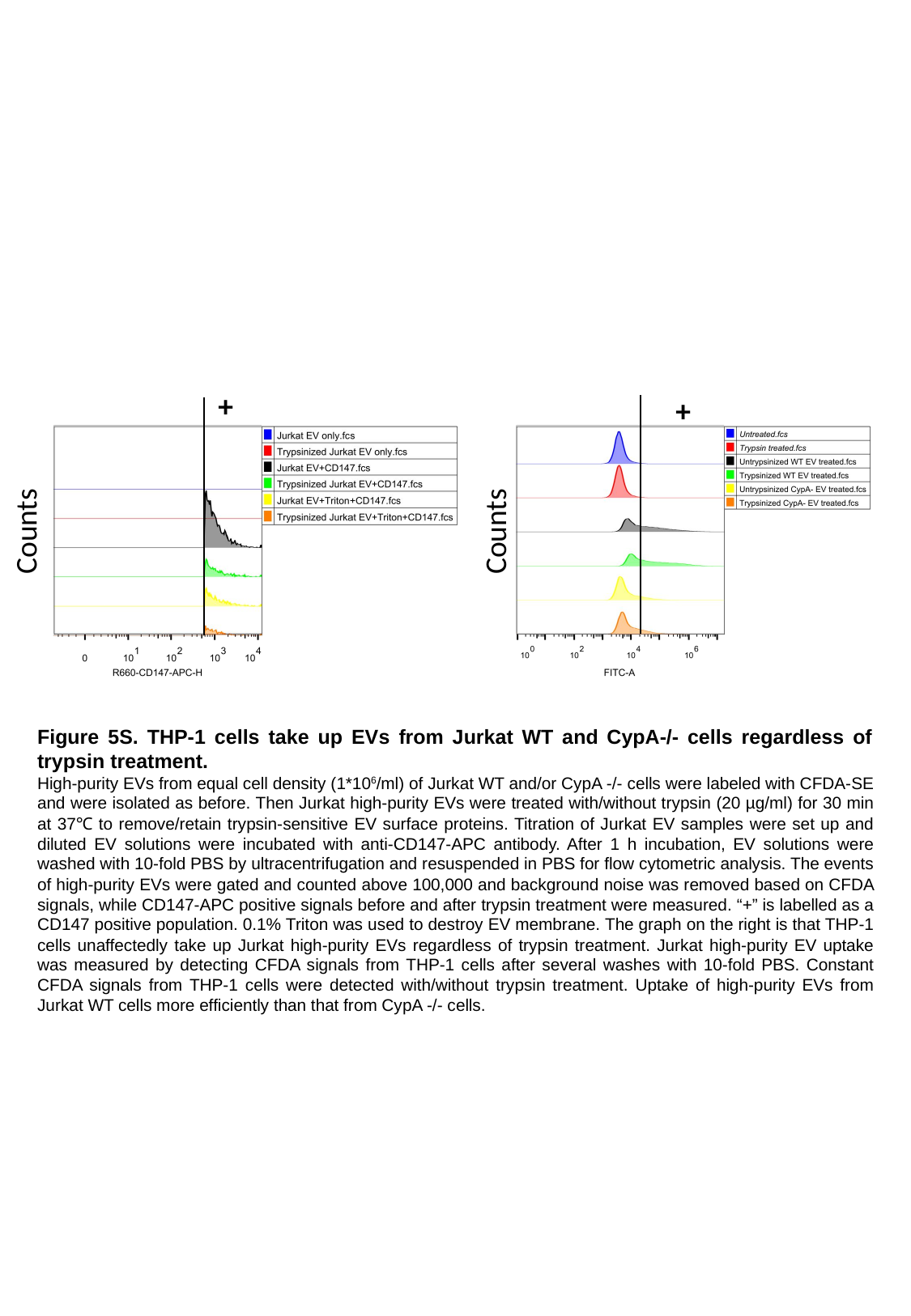

+
+
Counts
Counts
Figure 5S. THP-1 cells take up EVs from Jurkat WT and CypA-/- cells regardless of trypsin treatment.
High-purity EVs from equal cell density (1*106/ml) of Jurkat WT and/or CypA -/- cells were labeled with CFDA-SE and were isolated as before. Then Jurkat high-purity EVs were treated with/without trypsin (20 µg/ml) for 30 min at 37℃ to remove/retain trypsin-sensitive EV surface proteins. Titration of Jurkat EV samples were set up and diluted EV solutions were incubated with anti-CD147-APC antibody. After 1 h incubation, EV solutions were washed with 10-fold PBS by ultracentrifugation and resuspended in PBS for flow cytometric analysis. The events of high-purity EVs were gated and counted above 100,000 and background noise was removed based on CFDA signals, while CD147-APC positive signals before and after trypsin treatment were measured. “+” is labelled as a CD147 positive population. 0.1% Triton was used to destroy EV membrane. The graph on the right is that THP-1 cells unaffectedly take up Jurkat high-purity EVs regardless of trypsin treatment. Jurkat high-purity EV uptake was measured by detecting CFDA signals from THP-1 cells after several washes with 10-fold PBS. Constant CFDA signals from THP-1 cells were detected with/without trypsin treatment. Uptake of high-purity EVs from Jurkat WT cells more efficiently than that from CypA -/- cells.

## Slide 7
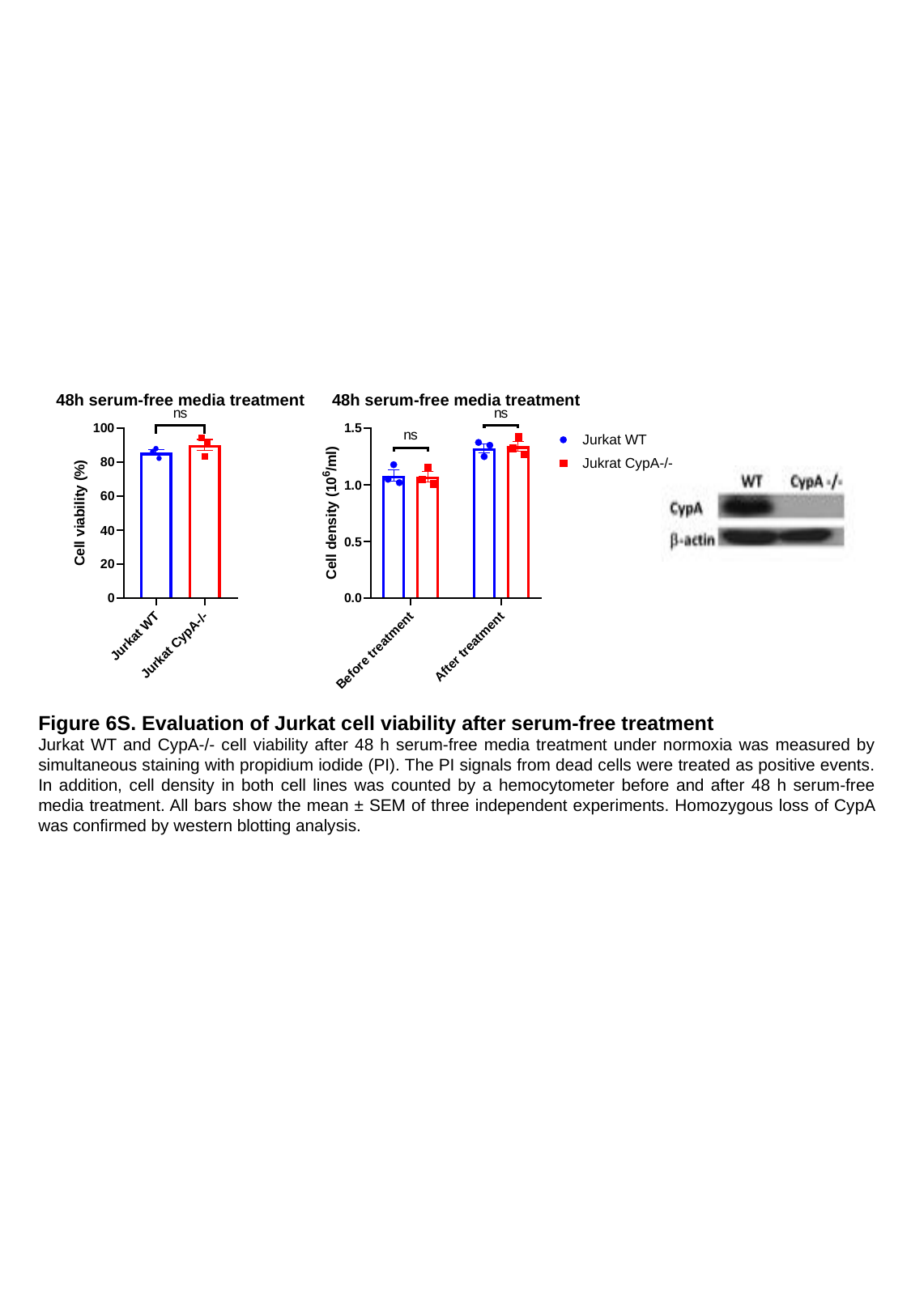

Figure 6S. Evaluation of Jurkat cell viability after serum-free treatment
Jurkat WT and CypA-/- cell viability after 48 h serum-free media treatment under normoxia was measured by simultaneous staining with propidium iodide (PI). The PI signals from dead cells were treated as positive events. In addition, cell density in both cell lines was counted by a hemocytometer before and after 48 h serum-free media treatment. All bars show the mean ± SEM of three independent experiments. Homozygous loss of CypA was confirmed by western blotting analysis.

## Slide 8
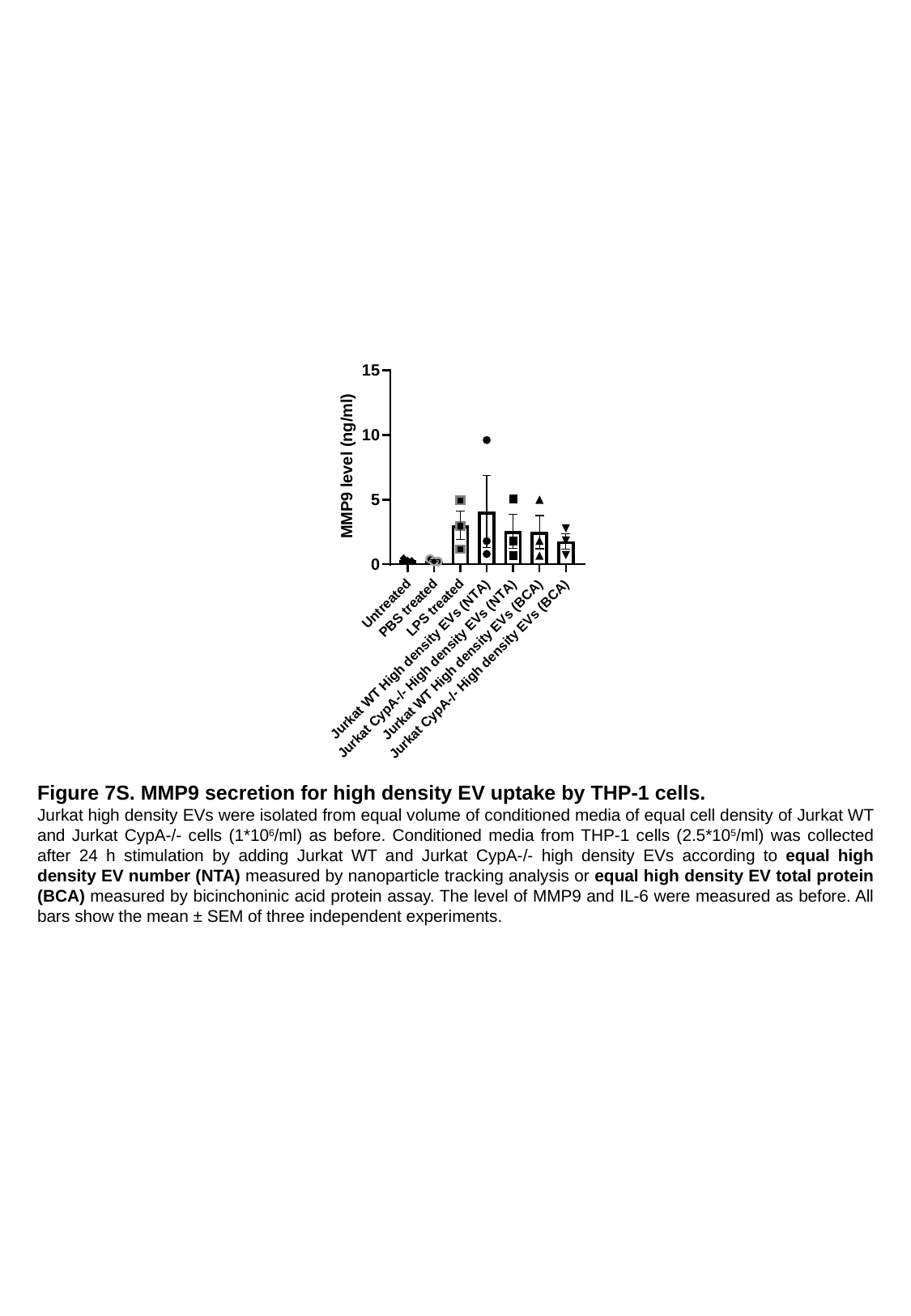

Figure 7S. MMP9 secretion for high density EV uptake by THP-1 cells.
Jurkat high density EVs were isolated from equal volume of conditioned media of equal cell density of Jurkat WT and Jurkat CypA-/- cells (1*106/ml) as before. Conditioned media from THP-1 cells (2.5*105/ml) was collected after 24 h stimulation by adding Jurkat WT and Jurkat CypA-/- high density EVs according to equal high density EV number (NTA) measured by nanoparticle tracking analysis or equal high density EV total protein (BCA) measured by bicinchoninic acid protein assay. The level of MMP9 and IL-6 were measured as before. All bars show the mean ± SEM of three independent experiments.

## Slide 9
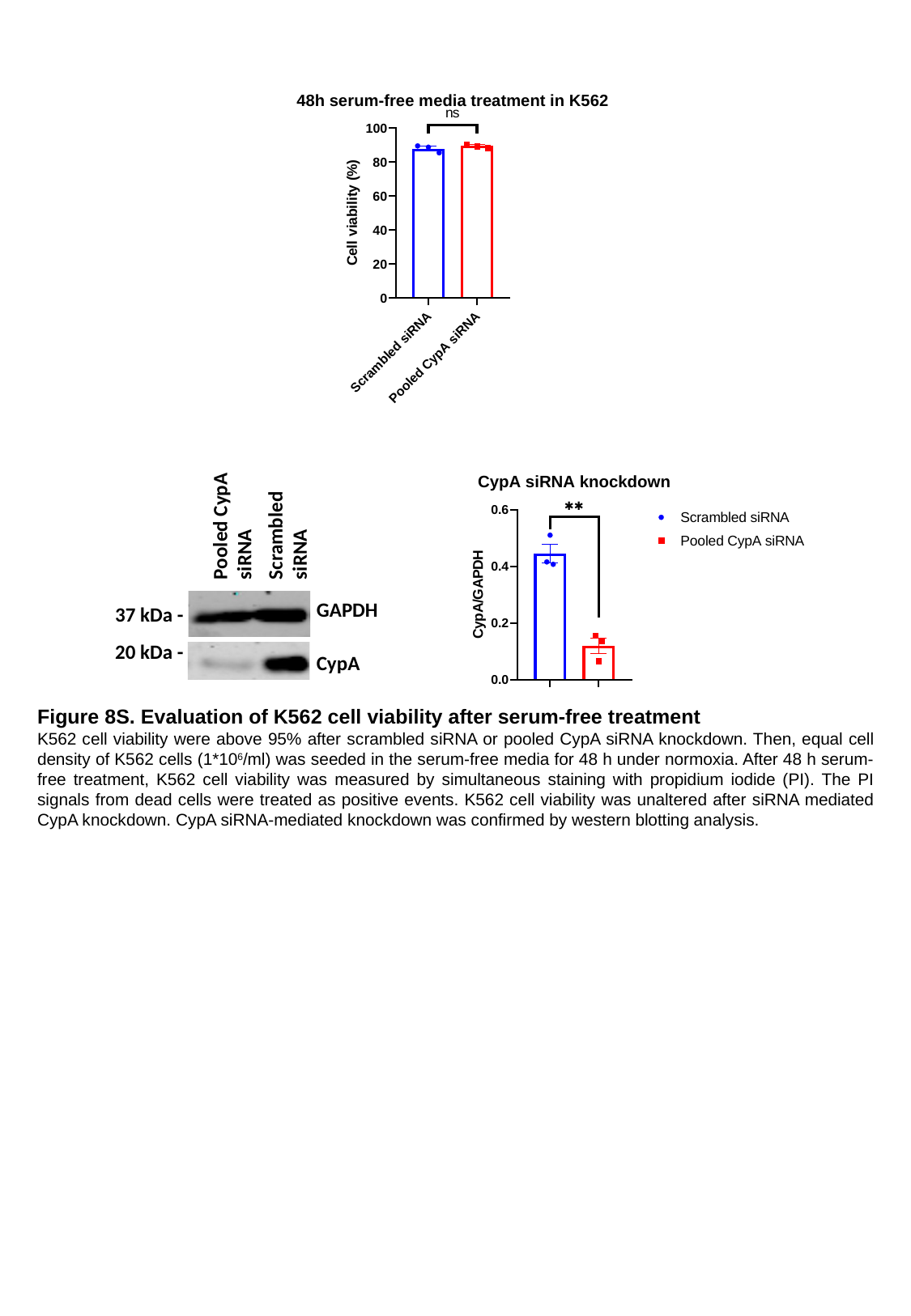

Pooled CypA siRNA
Scrambled siRNA
GAPDH
37 kDa -
20 kDa -
CypA
Figure 8S. Evaluation of K562 cell viability after serum-free treatment
K562 cell viability were above 95% after scrambled siRNA or pooled CypA siRNA knockdown. Then, equal cell density of K562 cells (1*106/ml) was seeded in the serum-free media for 48 h under normoxia. After 48 h serum-free treatment, K562 cell viability was measured by simultaneous staining with propidium iodide (PI). The PI signals from dead cells were treated as positive events. K562 cell viability was unaltered after siRNA mediated CypA knockdown. CypA siRNA-mediated knockdown was confirmed by western blotting analysis.

## Slide 10
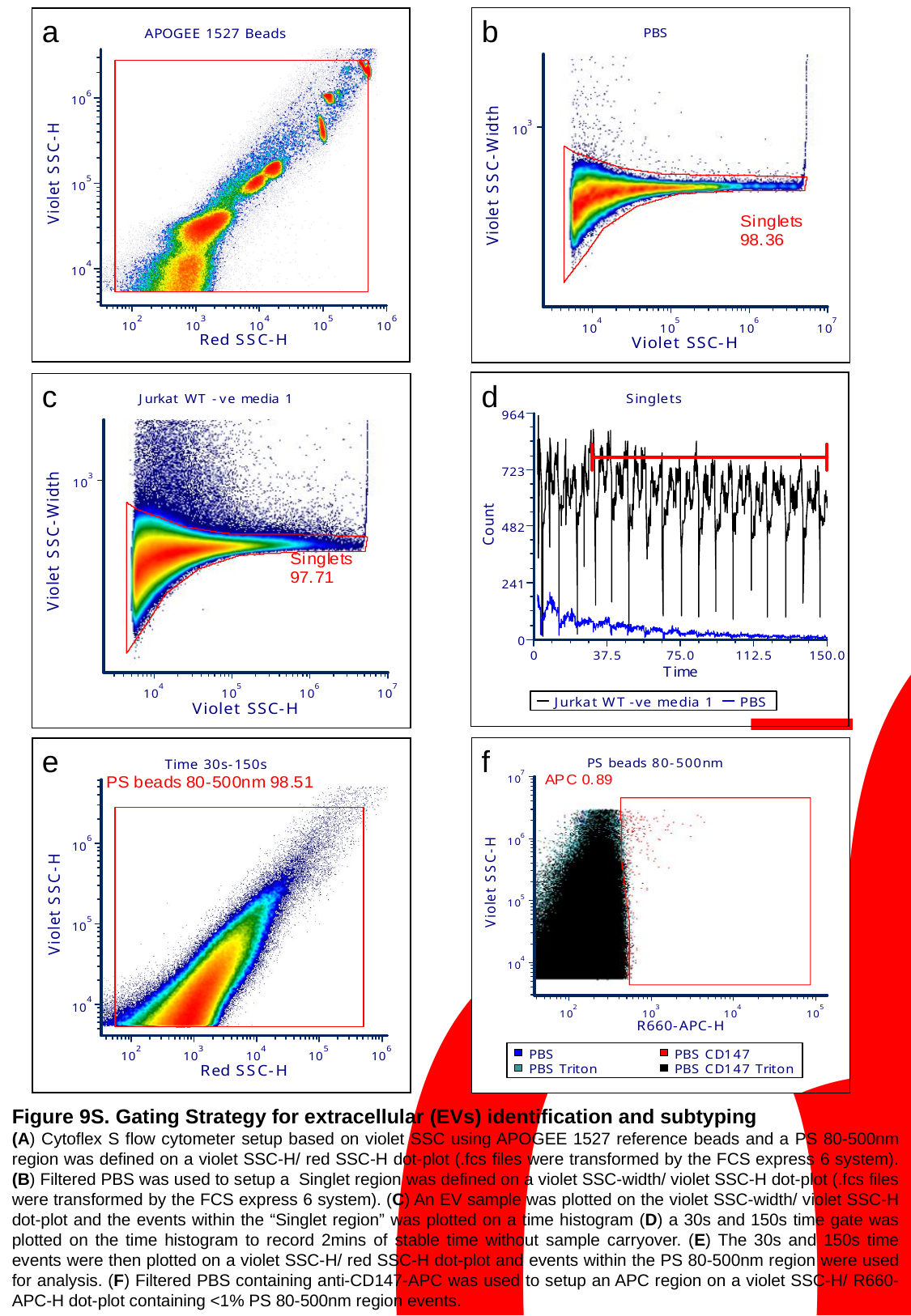

a
b
c
d
e
f
Figure 9S. Gating Strategy for extracellular (EVs) identification and subtyping
(A) Cytoflex S flow cytometer setup based on violet SSC using APOGEE 1527 reference beads and a PS 80-500nm region was defined on a violet SSC-H/ red SSC-H dot-plot (.fcs files were transformed by the FCS express 6 system). (B) Filtered PBS was used to setup a Singlet region was defined on a violet SSC-width/ violet SSC-H dot-plot (.fcs files were transformed by the FCS express 6 system). (C) An EV sample was plotted on the violet SSC-width/ violet SSC-H dot-plot and the events within the “Singlet region” was plotted on a time histogram (D) a 30s and 150s time gate was plotted on the time histogram to record 2mins of stable time without sample carryover. (E) The 30s and 150s time events were then plotted on a violet SSC-H/ red SSC-H dot-plot and events within the PS 80-500nm region were used for analysis. (F) Filtered PBS containing anti-CD147-APC was used to setup an APC region on a violet SSC-H/ R660-APC-H dot-plot containing <1% PS 80-500nm region events.

## Slide 11
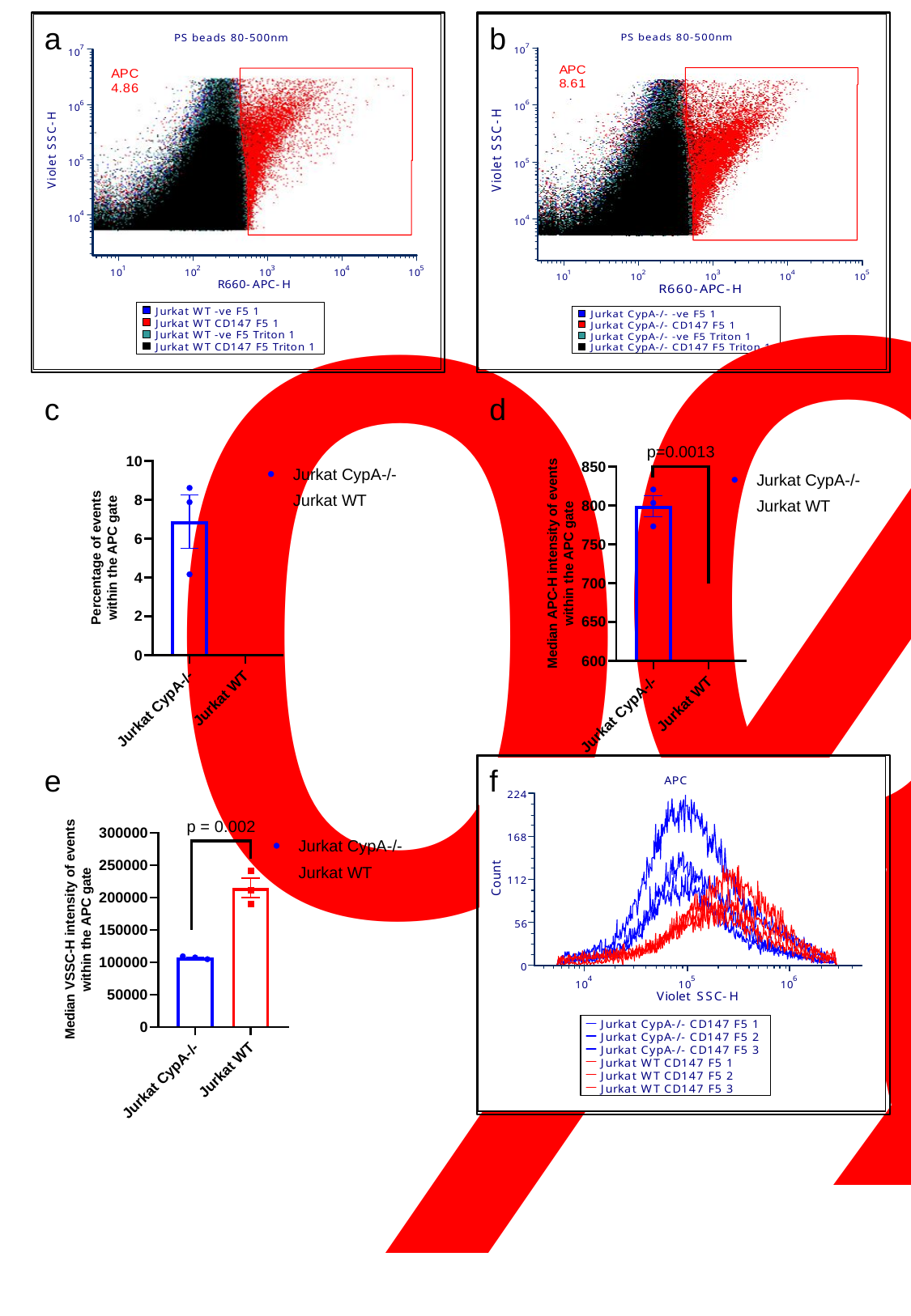

a
b
c
d
e
f

## Slide 12
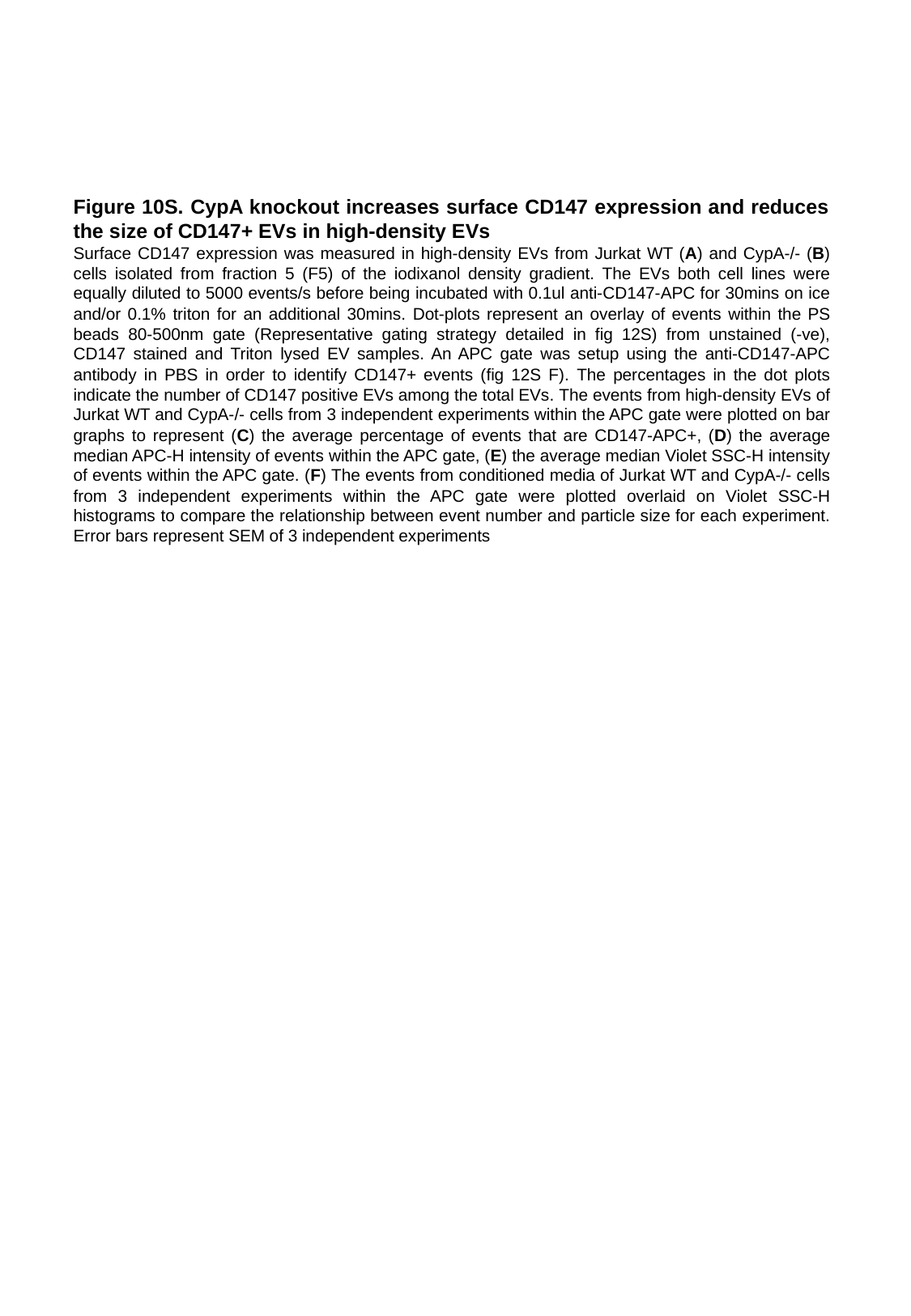

Figure 10S. CypA knockout increases surface CD147 expression and reduces the size of CD147+ EVs in high-density EVs
Surface CD147 expression was measured in high-density EVs from Jurkat WT (A) and CypA-/- (B) cells isolated from fraction 5 (F5) of the iodixanol density gradient. The EVs both cell lines were equally diluted to 5000 events/s before being incubated with 0.1ul anti-CD147-APC for 30mins on ice and/or 0.1% triton for an additional 30mins. Dot-plots represent an overlay of events within the PS beads 80-500nm gate (Representative gating strategy detailed in fig 12S) from unstained (-ve), CD147 stained and Triton lysed EV samples. An APC gate was setup using the anti-CD147-APC antibody in PBS in order to identify CD147+ events (fig 12S F). The percentages in the dot plots indicate the number of CD147 positive EVs among the total EVs. The events from high-density EVs of Jurkat WT and CypA-/- cells from 3 independent experiments within the APC gate were plotted on bar graphs to represent (C) the average percentage of events that are CD147-APC+, (D) the average median APC-H intensity of events within the APC gate, (E) the average median Violet SSC-H intensity of events within the APC gate. (F) The events from conditioned media of Jurkat WT and CypA-/- cells from 3 independent experiments within the APC gate were plotted overlaid on Violet SSC-H histograms to compare the relationship between event number and particle size for each experiment. Error bars represent SEM of 3 independent experiments

## Slide 13
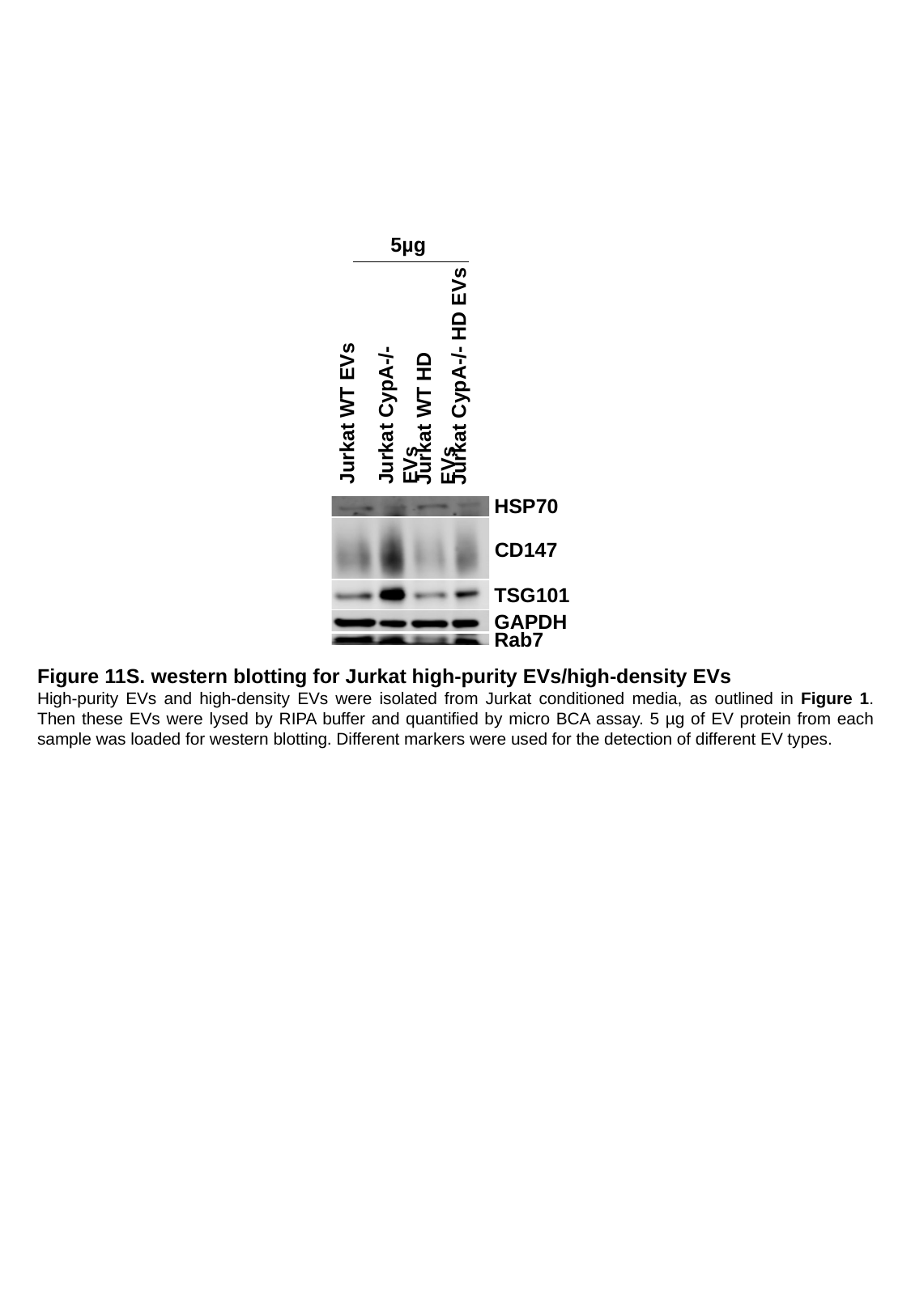

5µg
Jurkat CypA-/- HD EVs
Jurkat WT HD EVs
Jurkat CypA-/- EVs
Jurkat WT EVs
HSP70
CD147
TSG101
GAPDH
Rab7
Figure 11S. western blotting for Jurkat high-purity EVs/high-density EVs
High-purity EVs and high-density EVs were isolated from Jurkat conditioned media, as outlined in Figure 1. Then these EVs were lysed by RIPA buffer and quantified by micro BCA assay. 5 µg of EV protein from each sample was loaded for western blotting. Different markers were used for the detection of different EV types.

## Slide 14
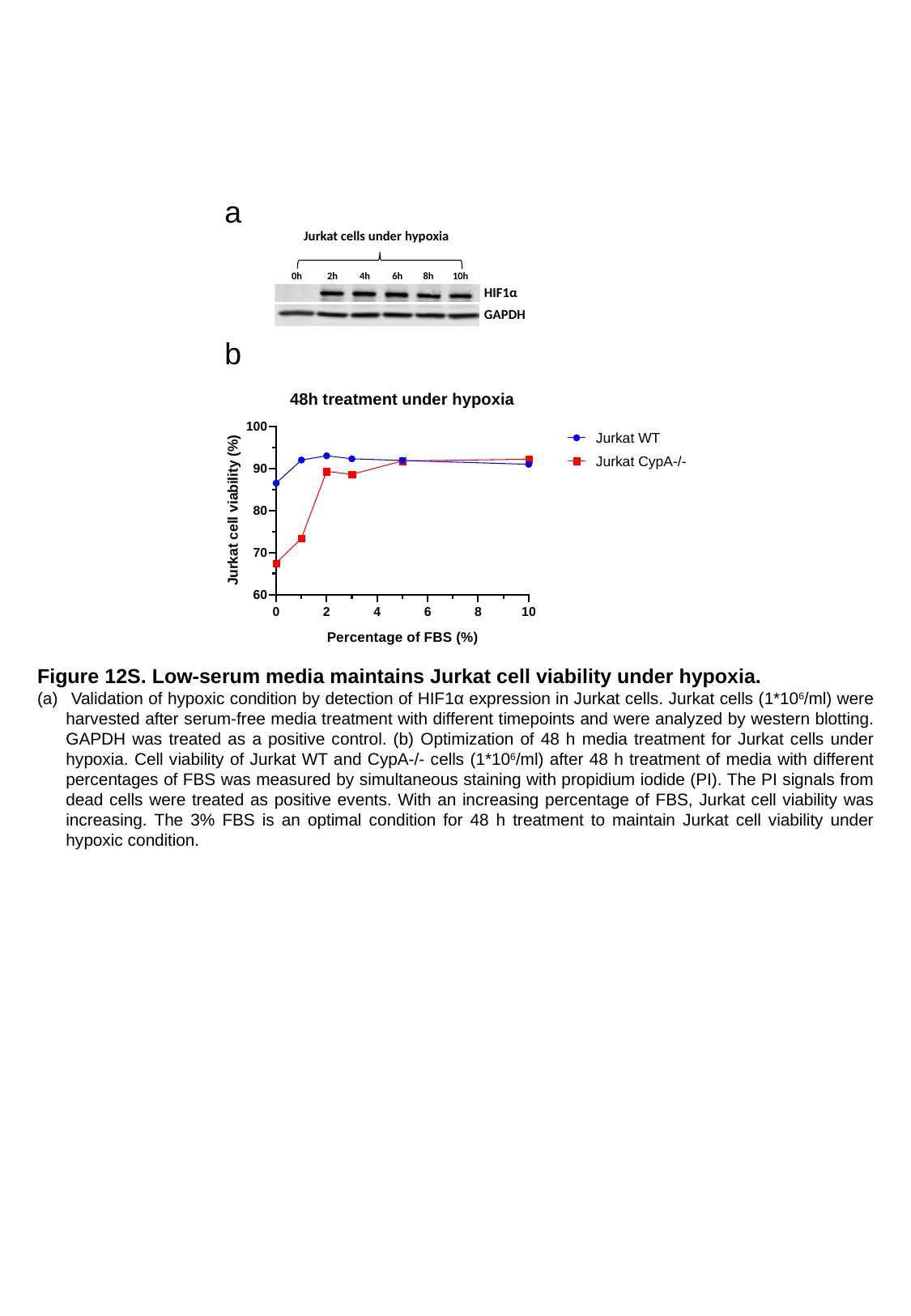

a
Jurkat cells under hypoxia
0h
2h
4h
6h
8h
10h
HIF1α
GAPDH
b
Figure 12S. Low-serum media maintains Jurkat cell viability under hypoxia.
 Validation of hypoxic condition by detection of HIF1α expression in Jurkat cells. Jurkat cells (1*106/ml) were harvested after serum-free media treatment with different timepoints and were analyzed by western blotting. GAPDH was treated as a positive control. (b) Optimization of 48 h media treatment for Jurkat cells under hypoxia. Cell viability of Jurkat WT and CypA-/- cells (1*106/ml) after 48 h treatment of media with different percentages of FBS was measured by simultaneous staining with propidium iodide (PI). The PI signals from dead cells were treated as positive events. With an increasing percentage of FBS, Jurkat cell viability was increasing. The 3% FBS is an optimal condition for 48 h treatment to maintain Jurkat cell viability under hypoxic condition.
