# Supplementary material for: Cyclophilin A regulates secretion of tumour-derived extracellular vesicles
Source: Transl Oncol. 2021 May 10;14(8):101112. doi: 10.1016/j.tranon.2021.101112 (PMC8131927; doi:10.1016/j.tranon.2021.101112)
Supplement: Supplementary file 2 [file mmc2.docx]

| **Filename** | **# of Events** | **APC Median** | **% of gated EVs** | **APC rSD** |
| --- | --- | --- | --- | --- |
| pbs -ve. | 4 | 482.3 | 0.01 | 17.72 |
| pbs CD147 | 250 | 549.9 | 0.89 | 106.82 |
| pbs -ve. triton | 216 | 484.05 | 0.03 | 47.07 |
| pbs CD147 triton | 128 | 499.8 | 0.02 | 53.15 |
| Jurkat CypA -/-1 CD147 media | 39532 | 850.5 | 8.64 | 370.21 |
| Jurkat CypA -/-2 CD147 media | 34492 | 858.2 | 7.44 | 381.77 |
| Jurkat CypA -/-3 CD147 media | 33392 | 892.9 | 6.4 | 428.32 |
| Jurkat WT1 CD147 media | 9820 | 624.7 | 2.24 | 123.5 |
| Jurkat WT2 CD147 media | 11828 | 624.35 | 2.39 | 124.46 |
| Jurkat WT3 CD147 media | 16935 | 731.2 | 3.03 | 261.09 |
| Jurkat CypA -/-1 -ve. media | 54 | 498.5 | 0.01 | 66.35 |
| Jurkat CypA -/-2 -ve. media | 53 | 484.5 | 0.01 | 56.19 |
| Jurkat CypA -/-3 -ve. media | 47 | 491.6 | 0.01 | 68.79 |
| Jurkat WT1 -ve. media | 41 | 507.5 | 0.01 | 64.05 |
| Jurkat WT2 -ve. media | 48 | 497 | 0.01 | 66.05 |
| Jurkat WT3 -ve. media | 48 | 498.75 | 0.01 | 73.02 |
| Jurkat CypA -/-1 CD147 media triton | 141 | 523.1 | 0.02 | 70.13 |
| Jurkat CypA -/-2 CD147 media triton | 91 | 555.2 | 0.01 | 71.02 |
| Jurkat CypA -/-3 CD147 media triton | 60 | 552 | 0.02 | 87.25 |
| Jurkat WT1 CD147 media triton | 91 | 605.9 | 0.03 | 204.9 |
| Jurkat WT2 CD147 media triton | 69 | 534.1 | 0.01 | 68.5 |
| Jurkat WT3 CD147 media triton | 73 | 546.9 | 0.02 | 59.3 |
| Jurkat CypA -/-1 -ve. media triton | 59 | 496.9 | 0.01 | 61.38 |
| Jurkat CypA -/-2 -ve. media triton | 74 | 514.5 | 0.01 | 74.87 |
| Jurkat CypA -/-3 -ve. media triton | 43 | 507 | 0.01 | 74.43 |
| Jurkat WT1 -ve. media triton | 30 | 495.6 | 0.01 | 54.34 |
| Jurkat WT2 -ve. media triton | 116 | 561.8 | 0.02 | 134.25 |
| Jurkat WT3 -ve. media triton | 31 | 504.8 | 0.01 | 74.43 |
| Jurkat CypA -/-1 CD147 f5 | 20572 | 773.3 | 8.61 | 291.63 |
| Jurkat CypA -/-2 CD147 f5 | 34869 | 820.8 | 7.88 | 354.93 |
| Jurkat CypA -/-3 CD147 f5 | 36153 | 803.5 | 4.16 | 357.31 |
| Jurkat WT1 CD147 f5 | 11920 | 679.8 | 4.86 | 200.97 |
| Jurkat WT2 CD147 f5 | 15227 | 648.3 | 2.74 | 180.43 |
| Jurkat WT3 CD147 f5 | 19868 | 646.1 | 2.63 | 187.25 |
| Jurkat CypA -/-1 -ve. f5 | 13 | 545.2 | 0.01 | 83.62 |
| Jurkat CypA -/-2 -ve. f5 | 61 | 476.4 | 0.02 | 34.69 |
| Jurkat CypA -/-3 -ve. f5 | 41 | 466.8 | 0.01 | 36.32 |
| Jurkat WT1 -ve. f5 | 24 | 477.4 | 0.01 | 36.69 |
| Jurkat WT2 -ve. f5 | 164 | 582.95 | 0.03 | 186.88 |
| Jurkat WT3 -ve. f5 | 59 | 483.7 | 0.01 | 43.59 |
| Jurkat CypA -/-1 CD147 f5 triton | 205 | 593.3 | 0.04 | 135.95 |
| Jurkat CypA -/-2 CD147 f5 triton | 128 | 583.25 | 0.03 | 124.32 |
| Jurkat CypA -/-3 CD147 f5 triton | 196 | 607.2 | 0.04 | 128.1 |
| Jurkat WT1 CD147 f5 triton | 97 | 558.6 | 0.02 | 95.18 |
| Jurkat WT2 CD147 f5 triton | 106 | 545.5 | 0.02 | 107.04 |
| Jurkat WT3 CD147 f5 triton | 134 | 547.5 | 0.02 | 87.47 |
| Jurkat CypA -/-1 -ve. f5 triton | 40 | 500.15 | 0.01 | 49.82 |
| Jurkat CypA -/-2 -ve. f5 triton | 43 | 504.2 | 0.01 | 67.01 |
| Jurkat CypA -/-3 -ve. f5 triton | 46 | 528.25 | 0.01 | 56.26 |
| Jurkat WT1 -ve. f5 triton | 59 | 506.6 | 0.01 | 69.09 |
| Jurkat WT2 -ve. f5 triton | 42 | 502.4 | 0.01 | 69.53 |
| Jurkat WT3 -ve. f5 triton | 30 | 504.5 | 0 | 63.97 |

Table S1. Flow cytometry statistics for EVs within the APC gate from all samples, highlighting the number of EVs within the gate, the % of EVs within the APC gate from the total PS bead 80-500nm gate and the median APC intensity and rSD.

| Filename | # of Events | VSSC-H Median | VSSC-H rSD |
| --- | --- | --- | --- |
| Jurkat CypA-/- CD147 media 1 | 39,532 | 74,615.5 | 65,335.36 |
| Jurkat CypA-/- CD147 media 2 | 34,492 | 81,219 | 68,019.98 |
| Jurkat CypA-/- CD147 media 3 | 33,392 | 60,721.85 | 53,277.23 |
| Jurkat CypA WT CD147 media 2 | 11,828 | 95,991.15 | 115,236.78 |
| Jurkat CypA WT CD147 media 3 | 16,935 | 148,537.91 | 129,214.23 |
| Jurkat CypA WT CD147 media 1 | 9,820 | 119,441.4 | 116,997.6 |
| Jurkat CypA-/- 1 CD147 f5 | 20,572 | 107,350.9 | 90,889.17 |
| Jurkat CypA-/- 2 CD147 f5 | 34,869 | 109,172.6 | 95,948.67 |
| Jurkat CypA-/- 3 CD147 F5 | 16,093 | 104,595.4 | 87,160.28 |
| Jurkat WT1 CD147 f5 | 11,920 | 190,353.34 | 179,940.85 |
| Jurkat WT2 CD147 f5 | 15,227 | 211,953.91 | 214,832.01 |
| Jurkat WT3 CD147 f5 | 19,868 | 242,061.25 | 232,374.35 |

Table S2. Flow cytometry statistics for EVs within the APC gate from all CD147-APC-stained samples, highlighting the number of EVs within the gate, and the median VSSC-H intensity and rSD.

| Filename | # of Events | VSSC-H Median | VSSC-H -rSD |
| --- | --- | --- | --- |
| Jurkat CypA-/- 1 CD147 media | 457,285 | 13,244.5 | 9,421.77 |
| Jurkat CypA-/- 2 CD147 media | 463,442 | 14,100.1 | 10,595.1 |
| Jurkat CypA-/- 3 CD147 media | 521,639 | 14,840.6 | 11,904.68 |
| Jurkat WT1 CD147 media | 439,157 | 13,591.2 | 9,717.85 |
| Jurkat WT2 CD147 media | 494,662 | 12,381.5 | 8,304.26 |
| Jurkat WT3 CD147 media | 558,906 | 14,229.1 | 10,891.77 |
| Jurkat CypA-/- 1 CD147 f5 | 239,005 | 16,031.3 | 13,660.23 |
| Jurkat CypA-/- 2 CD147 f5 | 442,222 | 21,965.25 | 21,793.11 |
| Jurkat CypA-/- 3 CD147 F5 | 474,596 | 20,179.45 | 19,389.81 |
| Jurkat WT1 CD147 f5 | 245,401 | 16,709.2 | 13,906.64 |
| Jurkat WT2 CD147 f5 | 555,422 | 16,539.9 | 13,975.21 |
| Jurkat WT3 CD147 f5 | 756,840 | 19,299.7 | 18,021.74 |

Table S3. Flow cytometry statistics for EVs within the PS bead 80-500nm gate from all CD147-APC-stained samples, highlighting the number of EVs within the gate, and the median VSSC-H intensity and rSD.

| **Requirement** | **Please Include Requested Information** |
| --- | --- |
| 1.1. Purpose | To compare the concentration of CD147+ EVs in Jurkat cell conditioned media between Jurkat WT and CypA-/- cells. |
| 1.2. Keywords | EV; extracellular vesicles, CypA; Cyclophilin A, CD147 |
| 1.3. Experiment variables | Jurkat cell conditioned media and Jurkat EVs isolated by density gradient ultracentrifugation from Jurkat WT and CypA-/- cells were measured from 3 independent isolations. Violet scatter-based triggering was used for the detection of particles (VSSC gain=300; VSSC-H threshold=5500). Parameters detected: violet side-scatter (VSSC), red side-scatter (RSSC), Allophycocyanin (APC) |
| 1.4. Organization name and address | Conway Institute, University College Dublin, Belfield, Dublin 4, Ireland |
| 1.5. Primary contact name and email address | Margaret McGee, margaret.mcgee@ucd.ie  Kieran Brennan, k.brennan@ucd.ie |
| 1.6. Date or time period of experiment | December 2020 – February 2021 |
| 1.7. Conclusions | CD147-APC+ EVs were detected in both WT and CypA-/- samples, with a higher percentage of CD147-APC+ EVs present in CypA-/- samples. The CD147-APC+ EVs present in CypA-/- samples had a higher median APC-H intensity and a lower VSCC-H than WT samples. |
| 1.8. Quality control measures | Daily calibration of the flow cytometer CytoFLEX Daily QC and CytoFLEX Daily IR QC Fluorospheres beads as per manufacturer specifications, followed by Apogee Mix 1527 Silica (Si) and polystyrene (PS) beads (Apogee Flow Systems Ltd. Hertfordshire, UK) were used in sizes of PS 80nm, PS 110nm, Si 180nm, Si 240nm, Si 300nm, PS 500nm, Si 590nm, Si 880nm and Si 1300nm |
| 2.1.1.1. (2.1.2.1., 2.1.3.1.) Sample description | Jurkat cell conditioned media and Jurkat EVs isolated by density gradient ultracentrifugation from Jurkat WT and CypA-/- cells were measured from 3 independent isolations. |
| 2.1.1.2. Biological sample source description | Conditioned media from T lymphocyte cell line Jurkat WT and CypA-/- cells were seeded at 1x 106 cells/ml in 20ml serum-free media in 145/20 mm cell culture dishes for the 48h serum-free media treatment. Conditioned media was used for EV isolation by density gradient ultracentrifugation – see Materials and Methods section for description. |
| 2.1.1.3. Biological sample source organism description | T lymphocyte cell line Jurkat WT and CypA-/-, CypA-/- cell line was produced by homologous recombination (Braaten and Jeremy Luban, 2001) |
| 2.1.2.2. Environmental sample location | NA |
| 2.3. Sample treatment description | Jurkat WT and CypA-/- cell conditioned media and isolated EVs in PBS were labelled with anti-human CD147 mouse-IgG1-APC (Clone: MEM-M6/1, Manufacturer: ThermoFisher, Cat No. A15706, Lot No. 76056999)). All samples were serially diluted from 1:2 to 1:500 prior to staining to achieve an event count of 5000 events/s (30,000 events/µl) and analyzed in PBS. |
| 2.4. Fluorescence reagent(s) description | anti-human CD147 mouse-IgG1-APC (Clone: MEM-M6/1, Manufacturer: ThermoFisher, Cat No. A15706, Lot No. 76056999)) was used to label Jurkat WT and CypA-/- cell conditioned media and isolated EVs. |
| 3.1. Instrument manufacturer | Beckman Coulter |
| 3.2. Instrument model | Beckman Coulter CytoFLEX LX Flow Cytometer |
| 3.3. Instrument configuration and settings | CytoFLEX LX (100mW 405nm, 50mW 488nm, 50mW 561nm, 50mW 638nm)  405 – 405/10 (V-SSC), 488 – 488SSC (B-SSC), 488 – 530/30 (Apogee beads FITC), 638 – 638/6 (R-SSC), 638 – 660/10 (APC) |
| 4.1. List-mode data files | FC files and the analysis workspace have been uploaded FlowRepository and can also be obtained by contacting the corresponding author. |
| 4.2. Compensation description | No compensation was needed |
| 4.3. Data transformation details | No transformation was needed |
| 4.4.1. Gate description | See Methods section and Figure 9S of manuscript |
| 4.4.2. Gate statistics | See Methods section, Figure 9S and Table S1, S2, and S3of manuscript |
| 4.4.3. Gate boundaries | See Methods section and Figures 9S of manuscript |

Table S4. Minimum Information about a Flow Cytometry Experiment (MIFlowCyt) checklist

| **Framework Criteria** | **Please complete each criterion** |
| --- | --- |
| 1.1 Preanalytical variables conforming to MISEV guidelines. | **Cell Culture**  The human B lymphocytic leukemia cell line HG3 and I83, human myelogenous leukemia cell line K562, myeloma cell line U266, T lymphocyte cell line Jurkat WT and CypA-/-, and human monocytic cell line THP-1 were grown in RPMI 1640 media (Gibco 61870010, with GlutaMAX) with 10% fetal bovine serum (FBS) (Gibco) and 1% Penicillin-Streptomycin (Gibco). RPMI 1640 media with 1% Penicillin-Streptomycin only was used as serum-free media. Cells were cultured at 37 ℃ in 5% CO2 humidified incubator and passaged every 2-3 days at 70%-80% confluence. All cell lines were checked quarterly for mycoplasma contamination  **Conditioned media preparation**  HG3, I83, Jurkat, K562, U266 cells were grown in T175 cell culture flasks and cell viability was more than 90% before washing the cells 3 times in serum-free media and seeding the cells at 1x 106 cells/ml in 20ml serum-free media in 145/20 mm cell culture dishes for the 48h serum-free media treatment.  Conditioned media from each cell line after 48 h serum-free medium treatment was used for EV isolation. Cell viability was more than 85% at the time of EV harvesting. Cells were removed by centrifugation at 400 g for 5 min, and the media was pipetted into a new tube and the cell debris were removed by centrifugation at 2000 g for 20 min. The media was pipetted into a new tube for ultracentrifugation.  **Ultracentrifugation (UC)**  Conditioned media after 2000 g centrifugation was centrifuged at 10,000 gavg for 35min to get the10K pellets, with the supernatant being pipetted into a new tube and centrifuged at 120,000 gavg for 3h 15min to get 120K pellets. Ultracentrifugation was performed with the SW32Ti rotor (k-factor 204, Beckman Coulter) and centrifugation durations adjusted based on a “50 nm cut-off size” as described in the paper [23]). The 10K and 120K pellets were subsequently washed in PBS and pelleted again by ultracentrifugation before being resuspended in 100µl PBS.  **Floatation density gradient (DG-UC)**  For density gradient ultracentrifugation, the conditioned media after 2000 g centrifugation was centrifuged at 120,000 gavg for 3h 15min to get 120K pellets. Ultracentrifugation was performed with the SW32Ti rotor (k-factor 204, Beckman Coulter) and centrifugation durations adjusted based on a “50 nm cut-off size” as described in the paper [23]). 2 methods of density gradients were used: one method to separate the total EV population from non EV protein (method A) and one method to separate EV populations based on EV density (Method B). For **method A**, the supernatant was pipetted off and the pellet was resuspended in 300 µl PBS and mixed with 2.7 ml 60% iodixanol and laid at the bottom of an SW41ti ultracentrifuge tube. 3ml of 40% iodixanol was overlaid on top followed PBS forming a discontinuous gradient. Ultracentrifugation was performed at 120,000 gavg with the SW41Ti rotor (k-factor 143.9, Beckman Coulter, stop without brake) for 16 h 15 min. The EV layer present between PBS and 40% iodixanol was transferred to a new tube and washed in PBS with ultracentrifugation at 120,000 gavg for 3 h 15 min.  For **method B**, the supernatant was pipetted off and the pellet was resuspended in 1.2ml PBS and mixed with 1.8 ml 60% iodixanol and laid at the bottom of an SW41ti ultracentrifuge tube. 3ml of 24% iodixanol was overlaid on top followed by 3ml of 20% iodixanol and 10% iodixanol forming a discontinuous gradient. Ultracentrifugation was performed at 120,000 gavg with the SW41Ti rotor (k-factor 143.9, Beckman Coulter, stop without brake) for 16 h 15 min. 100 µl fractions were collected from the top to bottom of the tube and distributed into 96-well plate for absorbance measurement at 340 nm by multi-well microplate spectrophotometer and density was calculated from an Iodixanol standard curve. Fractions with densities <1.06 g/ml, 1.06 - 1.09 g/ml, 1.09 - 1.12 g/ml, 1.12 - 1.15 g/ml, 1.15 - 1.18 g/ml and 1.18 - 1.21 g/ml were pooled to form six individual fractions respectively. The fractions were transferred to a new tube and washed in PBS with ultracentrifugation at 120,000 gavg using the SW41Ti rotor (k-factor 143.9, Beckman Coulter) for 3 h 15 min. All centrifugation were performed at 10 ℃. The fractionated EV pellets were resuspended in 100µl PBS and were freshly processed. |
| 1.2 Experimental design according to MIFlowCyt guidelines. | **1.1 Purpose:** To compare the concentration of CD147+ EVs in Jurkat cell conditioned media between Jurkat WT and CypA-/- cells.  **1.2 Keywords**: EV; extracellular vesicles, CypA; Cyclophilin A, CD147  **1.3 Experimental variables:** Jurkat cell conditioned media and Jurkat EVs isolated by density gradient ultracentrifugation from Jurkat WT and CypA-/- cells were measured from 3 independent isolations. Violet scatter-based triggering was used for the detection of particles (VSSC gain=300; VSSC-H threshold=5500). Parameters detected: forward scatter (FSC), blue side-scatter (SSC), violet side-scatter (VSSC), red side-scatter (RSSC), Allophycocyanin (APC) and FITC channel for instrument set up. |
| 2.1 Sample staining details | The presence of CD147 was determined using CD147 antibody staining. Samples were serially diluted from 1:2 to 1:500 to achieve an event count of 5000 events/s (30,000 events/µl) and that dilution was used for antibody labelling. EVs were labelled with 0.1ml anti-CD147-APC (MEM-M6/1, Thermo Scientific) in 100 µl PBS for 30 mins on ice and protected from light. |
| 2.2 Sample washing details | No washing steps were performed. |
| 2.3 Sample dilution details | Samples were serially diluted from 1:2 to 1:500 to achieve an event count of 5000 events/s (30,000 events/µl) and that dilution was used for antibody labelling. |
| 3.1 Buffer alone controls. | A buffer-only control of 0.22 μm-filtered DPBS (Gibco 14190144) was recorded at the same flow cytometer acquisition settings as all other samples, including triggering threshold, and flow rate. The buffer-only control had a count of ~100 events/s. |
| 3.2 Buffer with reagent controls. | A buffer with reagent control (PBS-anti-human CD147 mouse-IgG1-APC (Clone: MEM-M6/1, Manufacturer: ThermoFisher, Cat No. A15706, Lot No. 76056999)) was recorded at the same flow cytometer acquisition settings as all other samples, including triggering threshold, and flow rate. Buffer with reagent controls had an event rate of ~200 events/s. |
| 3.3 Unstained controls. | Unstained controls were measured at the same dilution as matched stained controls. Flow cytometer acquisition settings were maintained for all samples, including triggering threshold, and flow rate. |
| 3.4 Isotype controls. | Isotype control was not used. Unstained controls were instead used as a negative staining control |
| 3.5 Single-stained controls. | Not applicable, samples were either unstained or single stained anti-human CD147 mouse-IgG1-APC |
| 3.6 Procedural controls. | Controls and samples were acquired with the same gain settings, no compensation applied, low flow rate, stopping after recording the samples for 2 minutes. |
| 3.7 Serial dilutions. | Samples were serially diluted from 1:2 to 1:500 to achieve an event count of 5000 events/s (30,000 events/µl) and that dilution was used for antibody labelling. |
| 3.8. Detergent treated EV-samples | Stained and unstained samples, were treated with 0.1% Triton X-100 for 30 min at room temperature to test the lability of CD147-APC stained events. These measurements were used to compare CD147-APC stained samples not treated with detergent. |
| 4.1 Trigger Channel(s) and Threshold(s). | Based on the buffer alone control and Apogee Mix 1527 beads detection was triggered on the 405 nm laser (VSSC gain=300; VSSC-H threshold=5500) |
| 4.2 Flow Rate / Volumetric quantification. | The flow rate on the CytoFlex LX was calibrated with samples measured at 10 µl/min. |
| 4.3 Fluorescence Calibration. | Daily calibration of the flow cytometer CytoFLEX Daily QC and CytoFLEX Daily IR QC Fluorospheres beads (Beckman Coulter B53230 and C06147, respectively) as per manufacturer specifications. |
| 4.4 Light Scatter Calibration. | The gain of the three scatters was established with the help of the Apogee Mix beads (VSSC gain=300; VSSC-H threshold=5500), to minimize the noise and maximize the signal and trying to place the three scatters with very similar values |
| 5.1 EV diameter/surface area/volume approximation. | Not preformed |
| 5.2 EV refractive index approximation. | Not preformed |
| 5.3 EV epitope number approximation. | Not preformed |
| 6.1 Completion of MIFlowCyt checklist. | See attached MIFlowCyt Checklist |
| 6.2 Calibrated channel detection range | Not applicable |
| 6.3 EV number/concentration. | Not applicable |
| 6.4 EV brightness. | Not applicable |
| 7.1. Sharing of data to a public repository. | FC files and the analysis workspace have been uploaded FlowRepository and can also be obtained by contacting the corresponding author. |

Table S5. Minimum Information about a Flow Cytometry Experiment (MIFlowCyt) EV Reporting Framework
